# FAM171B stabilizes vimentin and enhances CCL2-mediated TAM infiltration to promote bladder cancer progression

**DOI:** 10.1186/s13046-023-02860-5

**Published:** 2023-11-02

**Authors:** Wei-Min Hu, Ming Li, Jin-Zhuo Ning, Yu-Qi Tang, Tian-Bao Song, Lin-Zhi Li, Fan Zou, Fan Cheng, Wei-Min Yu

**Affiliations:** Department of Urology, Renmin Hospital of Wuhan University, Wuhan, 430060 China

**Keywords:** Bladder cancer, CCL2, Macrophage, Vimentin

## Abstract

**Background:**

Invasion and metastasis are the main causes of unfavourable prognosis in patients diagnosed with bladder cancer. The efficacy of immunotherapy in bladder cancer remains suboptimal due to the presence of an immunosuppressive microenvironment. The novel protein family with sequence similarity 171B (FAM171B) has been identified, but its precise role and mechanism in bladder cancer remain unclear.

**Methods:**

In this study, we conducted an analysis to investigate the associations between FAM171B expression and the prognosis and clinicopathological stage of bladder cancer. To this end, we utilized RNA sequencing data from the TCGA and GEO databases, as well as tumor tissue specimens obtained from our clinical centre. RNA sequencing analysis allowed us to examine the biological function of FAM171B at the transcriptional level in bladder cancer cells. Additionally, we used immunoprecipitation and mass spectrometry to identify the protein that interacts with FAM171B in bladder cancer cells. The effects of FAM171B on modulating tumor-associated macrophages (TAMs) and vimentin-mediated tumor progression, as well as the underlying mechanisms, were clarified by phalloidin staining, immunofluorescence staining, ELISA, RNA immunoprecipitation, flow cytometry and a bladder cancer graft model.

**Results:**

FAM171B expression exhibits strong positive correlation with poor survival outcomes and advanced clinicopathological stages in patients with bladder cancer. FAM171B significantly promoted bladder cancer growth and metastasis, accompanied by TAM accumulation in the microenvironment, in vivo and in vitro. Through studies of the molecular mechanism, we found that FAM171B contributes to tumor progression by stabilizing vimentin in the cytoplasm. Additionally, our research revealed that FAM171B enhances the splicing of CCL2 mRNA by interacting with heterogeneous nuclear ribonucleoprotein U (HNRNPU), ultimately leading to increased recruitment and M2 polarization of TAMs.

**Conclusions:**

In this study, we identified FAM171B as a potent factor that promotes the progression of bladder cancer. These findings establish a solid theoretical foundation for considering FAM171B as a potential diagnostic and therapeutic biomarker for bladder cancer.

**Supplementary Information:**

The online version contains supplementary material available at 10.1186/s13046-023-02860-5.

## Introduction

Worldwide, bladder cancer is the second most prevalent urological malignancy and posed a severe threat to health [1]. Bladder urothelial carcinoma (BLCA) is the most common pathological type of bladder cancer, accounting for 90% of all bladder cancer. The poor prognosis of bladder cancer patients is attributed primarily to invasion and metastasis. While non-muscle-invasive bladder cancer (NMIBC) seldom metastasizes, approximately 15–20% of NMIBC cases progress to muscle-invasive bladder cancer (MIBC) [2]. MIBC has a high risk of metastasis, resulting in a considerably lower five-year survival rate than that of NMIBC. Notably, the advent of immunotherapy has resulted in promising survival benefits and revolutionized the treatment landscape for patients with advanced MIBC [3]. However, the management of MIBC remains challenging due to its persistently high recurrence rates despite treatment efforts [4]. Consequently, gaining a deeper understanding of the molecular mechanisms underlying bladder cancer progression is crucial for the development of novel therapeutic approaches.

Epithelial-to-mesenchymal transition (EMT) is the primary mechanism driving distant metastasis, the leading cause of mortality in bladder cancer patients [5]. EMT is a complex process wherein epithelial cells undergo a series of events to acquire mesenchymal traits, including enhanced motility and invasive properties [6]. Vimentin (VIM), a key marker of mesenchymal cells, has been found to be associated with tumor growth, invasion, and unfavourable prognosis in bladder cancer [7–9]. The upregulation of vimentin during EMT in bladder cancer cells implies that targeting vimentin has potential as a therapeutic strategy to impede the progression of metastatic disease.

The tumor microenvironment (TME) comprises a diverse array of stromal cell types that contribute to tumor progression [10]. Among these cells, tumor-associated macrophages (TAMs) constitute the most abundant immune cell population [11]. TAMs are a major obstacle to antitumor immunity and play a pivotal role in the failure of immunotherapy [12, 13]. The chemokine profile in the TME significantly influences the orientation and phenotype of macrophages. Notably, chemokine (C-C motif) ligand 2 (CCL2) plays a critical role in macrophage recruitment and is involved in cancer cell proliferation, cancer metastasis, and the establishment of an immunosuppressive TME [14, 15]. An understanding of the molecular mechanisms underlying the sustained expression of CCL2 in bladder cancer could provide a novel theoretical basis for the development of effective anti-CCL2 therapies.

FAM171B (family with sequence similarity 171B) is a widely expressed and evolutionarily conserved protein. The human and mouse FAM171B proteins possess similar domain structures. A group of researchers identified FAM171B as a candidate gene in a molecularly uncharacterized neurodegenerative disease, with its expression observed in the brain [16]. Additionally, analysis of RNA sequencing data has suggested that FAM171B might play a role in the progression of pulmonary arterial hypertension by stimulating immune infiltration and the immune response [17]. In human colorectal cancer cells, FAM171B has been identified as a functional target of miR-483-3p, involved in the regulation of oxaliplatin resistance [18]. However, despite these insights, our understanding of FAM171B’s structure, function, and specific role in cancer remains limited due to the scarcity of published research on the topic.

In the present study, we found that FAM171B expression was significantly positively associated with advanced clinicopathological stages and poor survival outcomes in bladder cancer patients. FAM171B significantly promoted bladder cancer growth and metastasis, accompanied by TAM accumulation in the microenvironment in vivo and in vitro. Through studies on the molecular mechanism, we found that FAM171B contributes to tumor progression by stabilizing vimentin in the cytoplasm. Additionally, our research revealed that FAM171B enhances the splicing of CCL2 mRNA by interacting with heterogeneous nuclear ribonucleoprotein U (HNRNPU), ultimately leading to increased recruitment and M2 polarization of TAMs. These findings provide new insights into the role of FAM171B in driving the progression of bladder cancer and shed light on the underlying molecular mechanisms.

## Materials and methods

### Patients and tissue microarray

A tissue microarray was constructed using tumor tissue samples from 70 patients who underwent transurethral resection or radical cystectomy for bladder urothelial carcinoma at Wuhan University People’s Hospital between 2020 and 2022. These patients had complete clinicopathological information and paraffin-embedded tissue specimens. The use of clinical samples in this study was approved by the Ethics Committee of Renmin Hospital of Wuhan University, and all procedures were conducted in accordance with ethical principles and local legislation.

### Bioinformatics analysis of the cancer genome atlas (TCGA) data and gene expression omnibus (GEO) data

We utilized a cohort of 425 human bladder specimens from the TCGA database, which included 406 BLCA samples and 19 paired paracancerous bladder tissues, to assess the expression levels of FAM171B in bladder cancer. The Kaplan-Meier method was used to examine the association between FAM171B expression and the survival outcomes (overall survival [OS], progression-free survival [PFS], disease-specific survival [DSS] and disease-free survival [DFS]) of BLCA patients. Furthermore, we validated the relationship between FAM171B expression and overall survival (OS) in BLCA using a separate cohort of 613 human BLCA specimens obtained from the Gene GEO database (GSE13507, GSE31684, GSE32894, GSE37815). Next, we analyzed the association of FAM171B expression with clinicopathological stage, chemokine expression and immune infiltration by TCGA-BLCA data. In addition, we performed pan-cancer prognostic analysis and pan-cancer immune analysis for FAM171B in 33 tumors in the TCGA database. The QUANTISEQ immune analysis algorithm represents a novel method for quantifying the fractions of immune cell types using bulk RNA-sequencing data, and it has undergone extensive validation in various tumor samples [19]. Chemotherapy response was predicted for TCGA-BLCA cohort based on the Genomics of Drug Sensitivity in Cancer (GDSC) database. The bioinformatics analysis and visualization of the results were performed using the R software.

### Cell culture and inhibitors

Human bladder cancer cell line T24 and human monocyte cell line THP-1 were cultured in 1640 medium supplemented with 10% fetal bovine serum. Mouse bladder cancer cell line MB49 and mouse macrophage cell line RAW264.7 were cultured in Dulbecco’s modified Eagle’s medium supplemented with 10% fetal bovine serum. To induce macrophage differentiation, THP-1 monocytes were incubated with Phorbol 12-myristate 13-acetate (PMA). Vimentin-IN-1 (inhibitor of vimentin), RS 504,393 (CCR2 inhibitor), and cycloheximide (protein synthesis inhibitor) were used in cellular experiments.

### Transfection

Flag-tagged FAM171B overexpression lentiviral vectors, shRNA targeting vectors, siRNAs targeting heterogeneous nuclear ribonucleoprotein U (HNRNPU) and HA-tagged HNRNPU/vimentin overexpression plasmids were custom-designed and synthesized by Genepharma (Shanghai, China) and Genomeditech (Shanghai, China). Transfection was conducted following established protocols. Cell lines with stable gene expression were selected using puromycin, and transfection efficiency was validated through Western blotting or quantitative real-time polymerase chain reaction (qRT-PCR) analyses.

### Cell proliferation assay and colony formation assay

The cell proliferation assay was estimated using the CCK-8 method. After incubated with CCK-8 solution for 2 h in a light-free environment, T24 and MB49 cells were measured the absorbance at 450 nm. In the colony formation assay, T24 and MB49 cells were seeded in six-well plates. After 21 days, the cells were fixed for 20 min and stained with crystal violet for visualization and counting.

### Phalloidin staining

After fixed and permeabilization, the actin cytoskeleton was stained using FITC-Phalloidin at room temperature for 1 h. Subsequently, the cells were stained with DAPI for 10 min. Fluorescence microscopy was employed to capture images.

### RNA immunoprecipitation (RIP) and qRT-PCR

Cells were transfected with the HNRNPU overexpression plasmid and subsequently subjected to RIP assay using the Magna RIP RNA-Binding Protein kit. Antibodies were included in the lysis buffer for immunoprecipitation, following the standard immunoprecipitation procedure. Protein A beads were then added to facilitate the immunoprecipitation of antibody-protein-RNA complexes. Finally, the RNA within the complexes was analyzed using qRT-PCR.

### Western blot

The western blot assay was performed as previously described. Antibody information: FAM171B (Novusbio, NBP1-93847); vimentin (Abmart, T55134); HNRNPU (Thermofisher, PA5-63604); ubiquitin (Proteintech, 10201-2); vimentin (Proteintech, 10366-1); E-cadherin (Proteintech, 20874-1); N-cadherin (Proteintech, 22018-1); HA (Abmart, M20003); DYKDDDDK (Abmart, M20008); GAPDH (Abmart, M20006); β-tubulin (Abmart, M20005); β-actin (Abmart, P30002).

### Immunofluorescent staining (IF), immunohistochemical staining (IHC) and hematoxylin-eosin staining (HE)

The immunofluorescent (IF) assay, immunohistochemical (IHC) assay, and hematoxylin-eosin (HE) assay were performed as previously described. Antibody information: FAM171B (Novusbio, NBP1-93847); vimentin (Abmart, T55134); HNRNPU (Thermofisher, PA5-63604); CD206 (Proteintech, 18704-1); CD8 (Proteintech, 66868-1); α-SMA (Proteintech, 14395-1); CD68 (Proteintech, 28058-1); F4/80 (Proteintech, 29414-1); ARG1 (Proteintech, 16001-1). For immunofluorescent colocalization analysis, the Colocalization Finder and Scatter J plugins of Image J were employed. The criteria for colocalization were set as follows: Pearson correlation coefficient (r) > 0.5 and Manders’ colocalization coefficients (M1 and M2) > 0.5.

### Coimmunoprecipitation assay and mass spectrometry

In brief, T24 cells overexpressing flag-tagged FAM171B and control T24 cells were harvested. The cell lysates were incubated with Anti-DYKDDDDK beads at 4 °C for 4 h. Following denaturing elution, the supernatant was subjected to western blot analysis using the specified antibodies. Successful immunoprecipitation was confirmed, and the remaining supernatant was utilized for mass spectrometry analysis at Bioprofile (Shanghai, China). The identified interacting proteins were further analyzed using protein-protein interaction (PPI) analysis through the STRING database. To validate protein interactions in the MB49 mouse bladder cancer cell line, co-transfected cells expressing Flag-tagged FAM171B and HA-tagged vimentin/HNRNPU were generated. Immunoprecipitation was carried out using anti-DYKDDDDK beads and anti-HA beads, following the same method as described above, and the resulting supernatant was analyzed by western blot using Anti-Flag and anti-HA antibodies. To validate the level of vimentin protein ubiquitination, we conducted an immunoprecipitation assay using vimentin antibodies (Proteintech, 10366-1) and protein A/G magnetic beads. The beads were mixed with vimentin antibodies, reaching a final concentration of 5 μg/mL, and incubated for 2 h. Subsequently, cell lysates were combined with the antibody-magnetic-bead complexes and incubated at 4 °C overnight. After elution, the resulting supernatant was subjected to western blot analysis. The Co-IP assay was performed using the IK-1011 Anti-DYKDDDDK (co-)immunoprecipitation kit, the IK-1009 Anti-HA (co-)immunoprecipitation kit and the IK-1004 Protein A/G (co-)immunoprecipitation kit (Bio-linkedin, Shanghai, China).

### Protein structure and protein docking

The protein structures of FAM171B and HNRNPU were derived from the AlphaFold Protein Structure Database. The vimentin structure was obtained from the Protein Data Bank (PDB: 1GK7). Molecular docking models of FAM171B-vimentin and FAM171B-HNRNPU were generated using GRAMM (Global RAnge Molecular Matching). The docking poses were evaluated based on their scores, and the top-scoring pose was chosen as the final conformation.

### Macrophage chemotaxis

Macrophage chemotaxis assay was analyzed using a 24-well transwell chamber equipped with polycarbonate membranes. RAW264.7 or THP-1 derived macrophages were seeded in the upper chambers, while MB49 or T24 cells were placed in the lower chamber. After incubating for 48 h, cells that migrated through the membranes were fixed and stained. The migrating cells in the upper chambers were then photographed and counted.

### Flow cytometry

The expression of macrophage phenotype-associated proteins was detected using flow cytometry. Following the antibody instructions, macrophages were incubated with the F4/80 antibody for 40 min. After a 15-minute fixation, macrophages cultured in vitro and cell suspensions from tumor samples were treated with a membrane breaker and CD68 and/or CD206 antibodies for 40 min. Subsequently, the cells were washed, resuspended, sorted using a Beckman Coulter CytoFLEX instrument, and analyzed with CytExpert software. The antibodies utilized in the flow cytometry experiments were procured from Elabscience.

### ELISA assay

The concentrations of IL-10, EGF, and CCL2 were determined using ELISA following the instructions. The ELISA kits utilized in the experiments were purchased from Thermo Fisher Scientific.

### Wound healing assay and transwell invasion assay

For the Wound Healing assay, T24 and MB49 cells were cultured in six-well plates until reaching 100% confluence. Subsequently, a sterile 200-μL pipette tip was used to create wounds in the cell monolayer. To minimize the impact of cell proliferation, the cells were cultured in serum-free medium. Photographic images of the wounds were taken at 0 and 48 h, using the six-well plates. For the Transwell invasion assay, a 24-well transwell chamber with matrigel was utilized. The upper chambers were loaded with cells and serum-free culture medium, while the lower chamber contained culture medium with 10% FBS. Cells that migrated through the membranes were fixed, stained, and then photographed and counted.

### RNA sequencing, data processing and gene difference analysis of T24 cells

Total RNA was isolated from both control and FAM171B-overexpressing T24 cells for RNA sequencing (RNAseq) analysis. The resulting library was subjected to sequencing using an Illumina Novaseq 6000 platform. Differentially expressed genes were determined based on a fold change greater than 2 or less than 0.5 and a p-value less than 0.01. Subsequently, Gene Ontology (GO), Kyoto Encyclopedia of Genes and Genomes (KEGG) pathway enrichment analyses and evolutionary gene genealogy Non-supervised Orthologous Groups (eggNOG) [20] functional analysis were conducted. All experimental protocols and data analyses were performed by Biomarker (Beijing, China).

### Animal experiments

Female BALB/c nude mice at 4 weeks of age were procured from Hunan Laboratory Animal Center. To establish the subcutaneous xenograft tumor model, 1 × 10^7^ MB49 mouse bladder cancer cells or T24 human bladder cancer cells in 0.1 ml physiological saline were injected subcutaneously into the flanks of the mice. In some groups, the mice were subjected to Vimentin-IN-1(10 mg/kg. p.o.), RS 504,393(5 mg/kg, i.v.) or normal saline treatment after 6 weeks. After two weeks of treatment, the mice were euthanized, and the tumors were dissected and weighed. The tumor volume (mm^3^) of both models was calculated as 0.5 × length × width^2^. This in vivo study was approved by the Ethics Committee of Wuhan University People’s Hospital.

### Statistical analysis

Student’s t-test, Pearson’s correlation test, Wilcoxon Rank Sum test and log-rank test were used to detect differences with GraphPad Prism version 9.20 software and R version 4.1.0. (* P < 0.05, **P < 0.01, ***P < 0.001) P values < 0.05 were regarded as statistically significant. All in vitro and in vivo experiments were performed independently at least three times.

## Results

### FAM171B expression is positively correlated with tumor progression and M2 TAM infiltration in BLCA patients

**Fig. 1:**
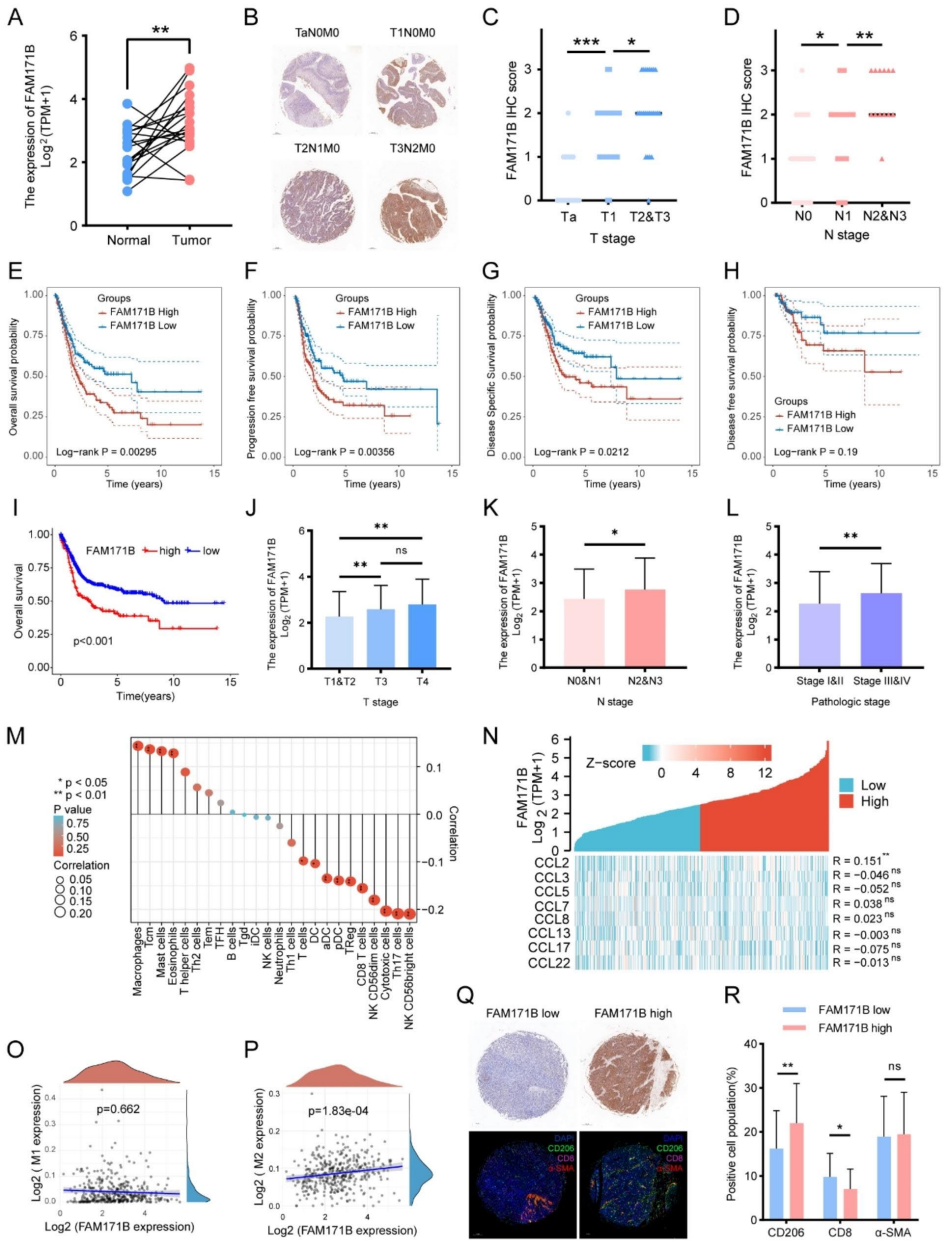
FAM171B expression is positively correlated with tumor progression and M2 TAM infiltration in BLCA patients. **A** Analysis of FAM171B expression in tumor tissue and paired normal tissue from TCGA dataset. **B** Representative IHC staining images of FAM171B in BLCA tissue array. **C** IHC score in different T stages from BLCA tissue array. **D** IHC score in different N stages from BLCA tissue array. **E** Relationship between FAM171B expression level and overall survival from TCGA dataset. **F** Relationship between FAM171B expression level and progression free survival from TCGA dataset. **G** Relationship between FAM171B expression level and disease specific survival from TCGA dataset. **H** Relationship between FAM171B expression level and disease-free survival from TCGA dataset. **I** Relationship between FAM171B expression level and overall survival from GEO dataset (GSE13507/GSE31684/GSE32894/GSE37815). **J** Analysis of FAM171B expression in different T stages from TCGA dataset. **K** Analysis of FAM171B expression in different N stages from TCGA dataset. **L** Analysis of FAM171B expression in different pathologic stages from TCGA dataset. **M** Lollipop plot demonstrating the correlation between FAM171B expression and different immune cell infiltration. **N** Heat map of the correlation between FAM171B expression and major monocyte/macrophage chemokines. **O** Immunoscore analysis of FAM171B and M1 macrophages in bladder cancer using QUANTISEQ algorithm. **P** Immunoscore analysis of FAM171B and M2 macrophages in bladder cancer using QUANTISEQ algorithm. **Q** Representative IHC staining images (FAM171B) and IF staining images (CD206, CD8 and α-SMA) in BLCA tissue array. **R** Proportion of immunofluorescence-positive cells (CD206, CD8 and α-SMA) in FAM171B high or low expression group from tissue array

To assess the clinical significance of FAM171B in bladder cancer, we conducted a comprehensive analysis using data from the TCGA database, the GEO database, and our clinical centre. Initially, we examined RNA sequencing data from 19 tumor tissues and matched paracancerous tissues in the TCGA-BLCA cohort. The results revealed higher expression levels of FAM171B in BLCA tumor tissues than the paired paracancerous tissues (Fig. 1A). Subsequently, we used immunohistochemical (IHC) staining on a tissue microarray containing clinical specimens from 70 BLCA patients to quantify FAM171B protein expression. Representative images are provided in Fig. 1B. Based on the clinical information obtained from the tissue microarray analysis and the results of IHC staining, we identified a significant correlation between a high FAM171B protein level and an advanced T stage (Fig. 1C) as well as an advanced N stage (Fig. 1D). Consistent results were obtained in analysis of RNA sequencing data from 406 BLCA tissues in the TCGA database (Fig. 1J, K and L). These findings suggest that increased FAM171B expression levels are associated with more advanced clinicopathologic grades and stages. Furthermore, our prognostic analysis demonstrated that BLCA patients with elevated FAM171B protein levels exhibited significantly lower overall survival (OS) (Fig. 1E), progression-free survival (PFS) (Fig. 1F), and disease-specific survival (DSS) rates (Fig. 1G). However, the difference in the disease-free survival (DFS) rate (Fig. 1H) was not statistically significant. To validate the relationships between FAM171B expression and survival rates in bladder cancer, we utilized an integrated cohort of 613 patients from datasets in the GEO database (GSE13507/GSE31684/GSE32894/GSE37815). The results demonstrated a significant association between an increased FAM171B protein level and a worse OS outcome in this cohort (Fig. 1I). Additionally, our pancancer prognostic analysis revealed a correlation between increased FAM171B expression and worse OS in 5 of the 33 cancers in the TCGA database (Fig. S1). Furthermore, analysis of immune cell infiltration in the TCGA-BLCA cohort indicated that FAM171B expression was most strongly correlated with macrophage infiltration (Fig. 1M). We further examined the relationship between the expression of chemokines affecting monocytes/macrophages and FAM171B. Among the evaluated chemokines, only CCL2 exhibited a statistically significant correlation with FAM171B (Fig. 1N). Regarding the association between FAM171B and the macrophage phenotype in BLCA, immunoassays based on the QUANTISEQ algorithm revealed a significant positive correlation between FAM171B expression and M2 macrophages (Fig. 1P), while no significant correlation with M1 macrophages was observed (Fig. 1O). Subsequently, we assessed the relationship between FAM171B and the infiltration of stromal cells (specifically, CD206^+^ M2 macrophages, CD8^+^ T cells and α-SMA^+^ fibroblasts) in the tissue microarrays. Representative images are shown in Fig. 1Q. Based on the results of IHC staining, we divided all samples into two groups: high expression and low expression of FAM171B. Through multiple immunofluorescence analyses, we discovered a significant positive correlation between the level of FAM171B protein and the infiltration of CD206^+^ M2 macrophages, while we observed a negative correlation between the level of FAM171B protein and the infiltration of CD8^+^ T cells. However, in terms of α-SMA^+^ fibroblast infiltration, we did not detect a significant difference between the high and low expression groups of FAM171B (Fig. 1R). Pancancer immune infiltration analysis indicated a positive association between FAM171B and M2 macrophage infiltration in 16 of 33 cancers in the TCGA database (Fig. S2). To investigate the impact of FAM171B on drug resistance in bladder cancer, we assessed the predicted chemotherapeutic response using the GDSC dataset. The findings revealed a significant reduction in drug sensitivity for Cisplatin, Mitomycin, Gemcitabine and Doxorubicin in the FAM171B high expression group within the TCGA-BLCA cohort. However, there was no statistically significant difference observed for Paclitaxel (Fig. S3). In conclusion, our findings indicate that FAM171B is associated with the infiltration of M2 macrophages and may serve as a potential prognostic indicator in bladder cancer.

### FAM171B promotes the malignant phenotype of bladder cancer cells

**Fig. 2:**
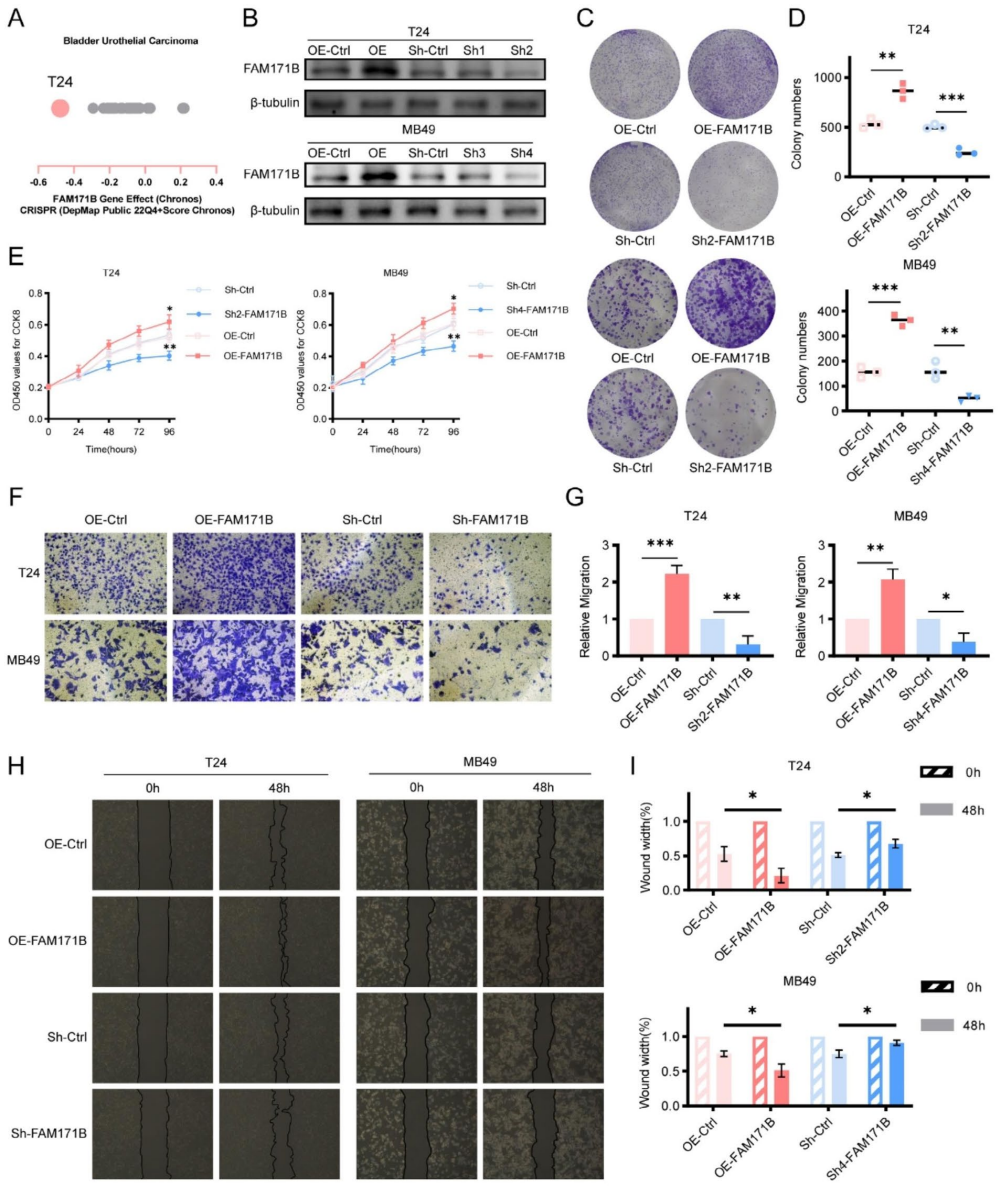
FAM171B promotes the malignant phenotype of bladder cancer cells. **A** The Chronos dependency score analysis of FAM171B in the 28 human bladder urothelial carcinoma cell lines in the CCLE database. The Chronos dependency score is based on data from a cell depletion assay. A lower Chronos score indicates a higher likelihood that the gene of interest is essential in a given cell line. **B** Protein levels of FAM171B in the knockdown and overexpression cell lines. **C** Representative colony formation images in different groups. **D** Colony formation analysis of T24 and MB49 cells after knockdown and overexpression of FAM171B. **E** Cell viability of T24 and MB49 after knockdown and overexpression of FAM171B. **F** Representative transwell images of T24 and MB49 cells after knockdown and overexpression of FAM171B. **G** Statistical results of the number of invasive cells in each group of T24 and MB49 cells. **H** Representative images of migration in T24 and MB49 cells after knockdown and overexpression of FAM171B. **I** Statistical results of the number of migrated cells in each group of T24 and MB49 cells

The Chronos dependency score revealed that the T24 cell line exhibited the lowest score among the 28 human bladder urothelial carcinoma cell lines in the CCLE database, indicating that FAM171B is more crucial in T24 cells than in the other cell lines (Fig. 2A). In addition to T24 human bladder urothelial carcinoma cells, we also included MB49 mouse bladder urothelial carcinoma cells in our study. To investigate the molecular function of FAM171B in bladder cancer cells, we knocked down and overexpressed FAM171B in T24 and MB49 cells and confirmed the efficiency through Western blot analysis (Fig. 2B). Subsequently, we conducted a CCK-8 assay to evaluate the impact of FAM171B on the viability of T24 and MB49 cells. The results in both cell lines demonstrated an increase in viability in FAM171B overexpressing cells and a decrease in viability in FAM171B knockdown cells after 96 h (Fig. 2E). Additionally, the results of the colony formation assay indicated a significant increase in the colony-forming ability of T24 and MB49 cells following FAM171B overexpression, while knockdown of FAM171B resulted in a decrease in the colony-forming ability (Fig. 2C and D). These in vitro findings suggest that the expression of the FAM171B gene influences cell proliferation, although the difference in cell viability becomes statistically significant only after 96 h. Furthermore, we aimed to elucidate the impact of FAM171B on bladder cancer cell metastasis using wound healing and transwell invasion. The results of the transwell invasion assay revealed reductions in the number of migrated cells in the lower chamber in the FAM171B knockdown T24 and MB49 cell groups compared to the corresponding control groups, while an increased number of migrated cells was observed in the lower chamber when FAM171B was overexpressed (Fig. 2F and G). Similarly, knockdown of FAM171B diminished the wound healing efficacy of T24 and MB49 cells, whereas FAM171B overexpression significantly accelerated cell migration (Fig. 2H and I). These in vitro results strongly suggest that FAM171B promotes the malignant phenotype of bladder cancer cells.

### RNA sequencing of T24 cells to investigate the function of FAM171B

**Fig. 3:**
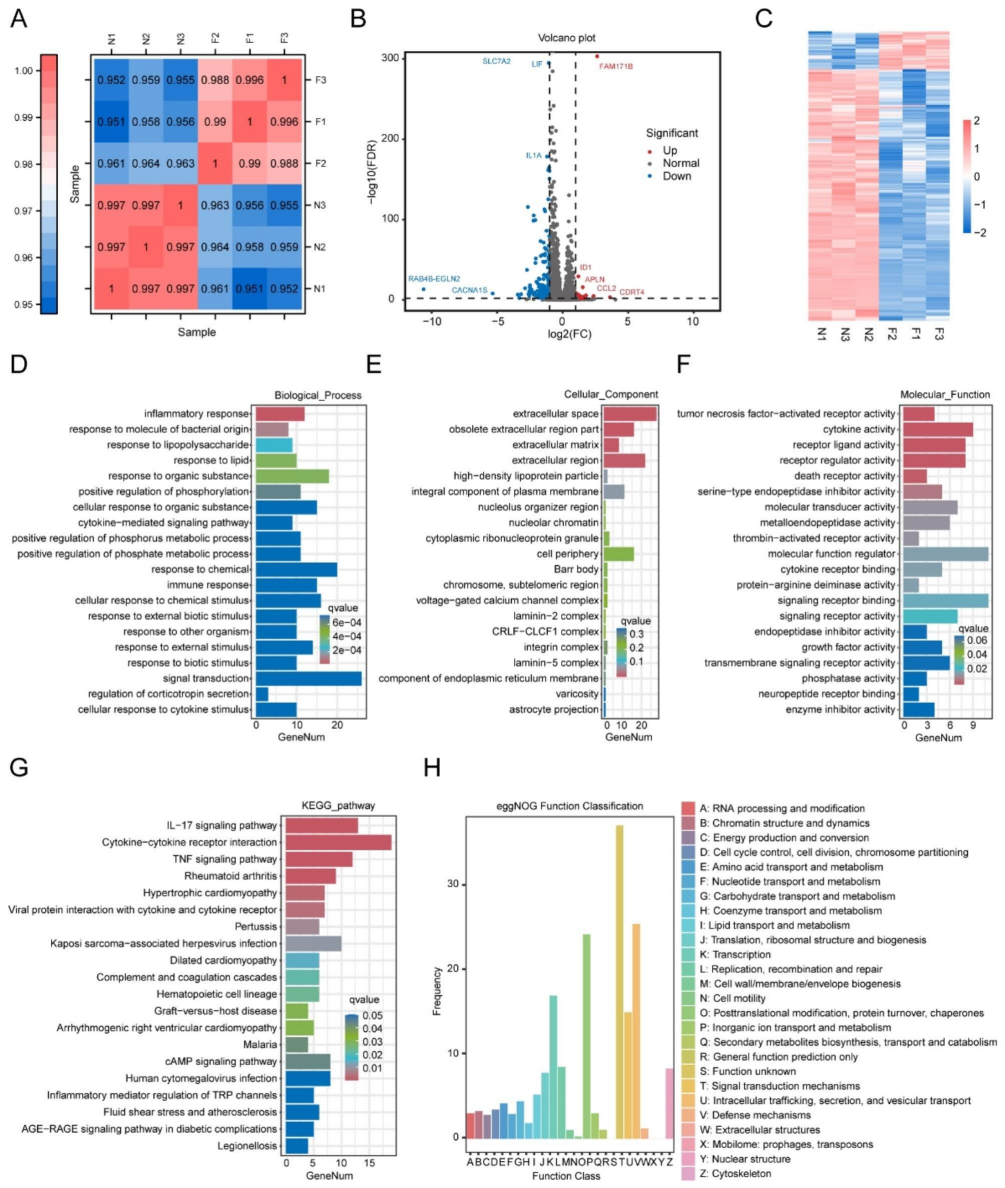
RNA sequencing of T24 cells to investigate the function of FAM171B. **A** Heatmap of the difference in overall mRNA expression between the FAM171B overexpression T24 cells (F1, F2 and F3) and the control T24 cells (N1, N2 and N3). The Pearson’s correlation coefficient was represented by a color scale. **B** Volcano plot of the differential gene expression between the FAM171B overexpression group and the control group. The vertical gray lines corresponded to two-fold up- and down-regulation, and the horizontal gray line represented a p value of 0.01. The red points represented significantly up-regulated gene, and the blue points represented significantly down-regulated gene. **C** Heatmap of specific differential genes between the FAM171B overexpression T24 cells and the control cells. **D, E**, **F** Histogram of GO annotations for the differential genes in biological processes (D), cellular components (E) and molecular functions (F). X axis represented the number of genes. Y axis showed the name of GO terms. **G** Histogram of KEGG analysis for the differential genes. X axis represented the number of genes. Y axis showed the name of enriched pathways. The p-value was represented by a color scale. The statistical significance increased from purple (relatively lower significance) to orange (relatively higher significance). **H** Histogram of eggNOG functional classification statistics for differentially expressed genes. X axis represented the function of the gene. Y axis showed the frequency (the number of genes)

To investigate the role of FAM171B in bladder cancer cells, we conducted RNA sequencing on FAM171B-overexpression and control T24 cells. The heatmap of intersample correlations demonstrated significant differences in mRNA expression levels between the FAM171B overexpression and control groups, whereas the differences within each group were relatively minor (Fig. 3A). Through analysis of the sequencing data, we identified 244 differentially expressed genes, defined as those with log2(FC) > 2 or log2(FC) < -2 and p < 0.01. The volcano plot shows some of the differentially expressed genes; upregulated genes included CDRT4, APLN and CCL2, and downregulated genes included RAB4B, CACNA1S and IL1A (Fig. 3B). Furthermore, the heatmap of gene expression across the six samples demonstrated significant differences in the expression of the differentially expressed genes among subgroups (Fig. 3C). To gain further insights into the specific function of FAM171B in T24 cells, we performed GO analysis, KEGG analysis, and eggNOG analysis. In the T24 cell line, GO analysis revealed significant associations of FAM171B with biological processes such as inflammatory response and cytokine-mediated signalling pathway (Fig. 3D). In the cellular component category, FAM171B was notably correlated with extracellular space and obsolete extracellular region parts (Fig. 3E). Regarding molecular functions, FAM171B showed significant associations with tumor necrosis factor-activated receptor activity and cytokine activity (Fig. 3F). KEGG analysis indicated that FAM171B was associated primarily with IL-17 signalling, cytokine-cytokine receptor interaction, and the TNF signalling pathway (Fig. 3G). Furthermore, eggNOG functional analysis revealed that FAM171B was predominantly involved in signal transduction mechanisms, replication, recombination, repair, and cytoskeleton-related functions (Fig. 3H). In summary, the results of RNA sequencing provided comprehensive insights into the diverse functions of FAM171B in the T24 human bladder cancer cell line, indicating that its functions primarily involve cytokines, signalling pathway transduction, and cytoskeleton-related processes.

### FAM171B interacts with vimentin and HNRNPU in bladder cancer cells

**Fig. 4:**
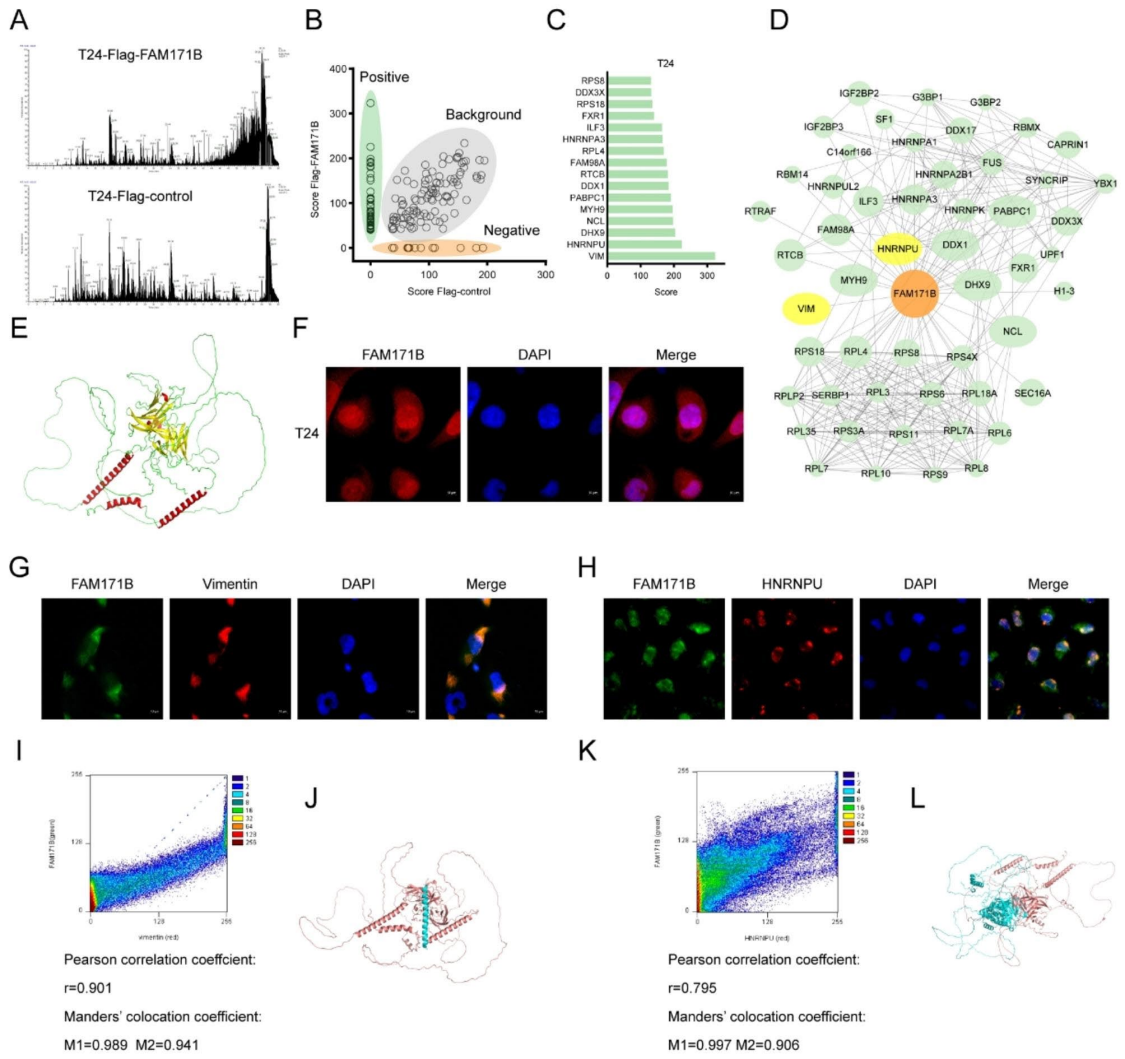
FAM171B interacts with vimentin and HNRNPU in bladder cancer cells. **A** Mass spectrometry analysis of interacting proteins enriched by immunoprecipitation. Immunoprecipitation is performed by Anti-DYKDDDDK beads on T24 cells transfected with FLAG-FAM171B or FLAG-control. **B** Analysis of positive and background proteins based on Mass Spectrometry scores. **C** The top 16 proteins with the highest scores based on MS results. **D** Protein interaction network map of proteins bound to FAM171B based on MS results. The protein interactions data is from STRING database. **E** The predicted protein structure image of FAM171B based on the Alpha Fold. **F** Immunofluorescence analysis images revealed the localization of FAM171B in T24 cells. **G** Colocalization of FAM171B and vimentin was visualized by confocal microscope in T24 cells. Cytoplasmic staining of FAM171B and vimentin was mostly merged together. **H** Colocalization of FAM171B and HNRNPU was visualized by confocal microscope in T24 cells. Nuclear staining of FAM171B and HNRNPU was mostly merged together. **I** ImageJ was used for colocation analysis of FAM171B and vimentin. Pearson correlation analysis showed that r > 0.5. Manders’ colocation coefficient showed that M1 > 0.5 and M2 > 0.5. **J** Computational docking model between FAM171B and vimentin. **K** ImageJ was used for colocation analysis of FAM171B and HNRNPU. Pearson correlation analysis showed that r > 0.5. Manders’ colocation coefficient showed that M1 > 0.5 and M2 > 0.5. **L** Computational docking model between FAM171B and HNRNPU

To elucidate the molecular role of FAM171B at the protein level, we conducted immunoprecipitation followed by mass spectrometry to identify the major interacting proteins of FAM171B in bladder cancer cells. The supernatant containing FAM171B-interacting proteins was collected using anti-DYKDDDDK beads from T24 cells overexpressing flag-tagged FAM171B and control cells. Subsequently, mass spectrometry was employed to identify specific positive interacting proteins (Fig. 4A and B). The interacting proteins with the highest scores, as determined by mass spectrometry, are indicated in Fig. 4C. Validated interactions between FAM171B and its interacting proteins were obtained using the STRING database. Subsequently, a protein interaction network for FAM171B was constructed, as shown in Fig. 4D. Among these proteins, VIM and HNRNPU, which exhibited the highest scores, were identified as key proteins interacting with FAM171B in T24 cells. The predicted structure of the human FAM171B protein was obtained from the AlphaFold website (Fig. 4E). To further explore the protein-protein interactions, we conducted simulations to visualize the docking modes of FAM171B with vimentin (Fig. 4J) and HNRNPU (Fig. 4L). The immunofluorescence results demonstrated the expression of the FAM171B protein in both the cytoplasm and nucleus of T24 cells (Fig. 4F). Double immunofluorescence staining and colocalization analysis were performed to validate the interactions between FAM171B and vimentin/HNRNPU. The results of double immunofluorescence staining indicated that the FAM171B-vimentin complex was localized primarily in the cytoplasm (Fig. 4G), whereas the FAM171B- HNRNPU complex localized predominantly in the nucleus (Fig. 4H). Colocalization analysis performed using Image J software confirmed the spatial coexpression of FAM171B with vimentin (Fig. 4I) (Pearson correlation coefficient r = 0.901; Manders’ co-location coefficient M1 = 0.989 and M2 = 0.941) and HNRNPU (Fig. 4K) (Pearson correlation coefficient r = 0.795; Manders’ co-location coefficient M1 = 0.997 and M2 = 0.906). The colocalization criteria for two proteins were defined as follows: Pearson correlation coefficient r > 0.5 and Manders’ co-location coefficient M1 > 0.5, M2 > 0.5 [21]. Moreover, we validated the interactions between FAM171B and vimentin/HNRNPU in MB49 bladder cancer cells from mice. Similar to the findings in human T24 cells, the FAM171B-vimentin complex was localized in the cytoplasm in MB49 cells (Figure S4A, S4C) (Pearson correlation coefficient r = 0.841; Manders’ co-location coefficient M1 = 0.981 and M2 = 0.950), while the FAM171B-HNRNPU complex was localized in the nucleus (Figure S4B, S4D) (Pearson correlation coefficient r = 0.764; Manders’ co-location coefficient M1 > 0.985 and M2 > 0.963). Additionally, we generated MB49 cells with combined overexpression of flag-tagged FAM171B and HA-tagged vimentin/HNRNPU. Immunoprecipitation using anti-DYKDDDDK and anti-HA beads confirmed the interactions of FAM171B with vimentin (Figure S4E) and HNRNPU (Figure S4F) in MB49 mouse bladder cancer cells. Collectively, these findings highlight vimentin and HNRNPU as the primary interacting proteins of FAM171B in bladder cancer cells.

### FAM171B stabilizes vimentin to promote the progression of bladder cancer

**Fig. 5:**
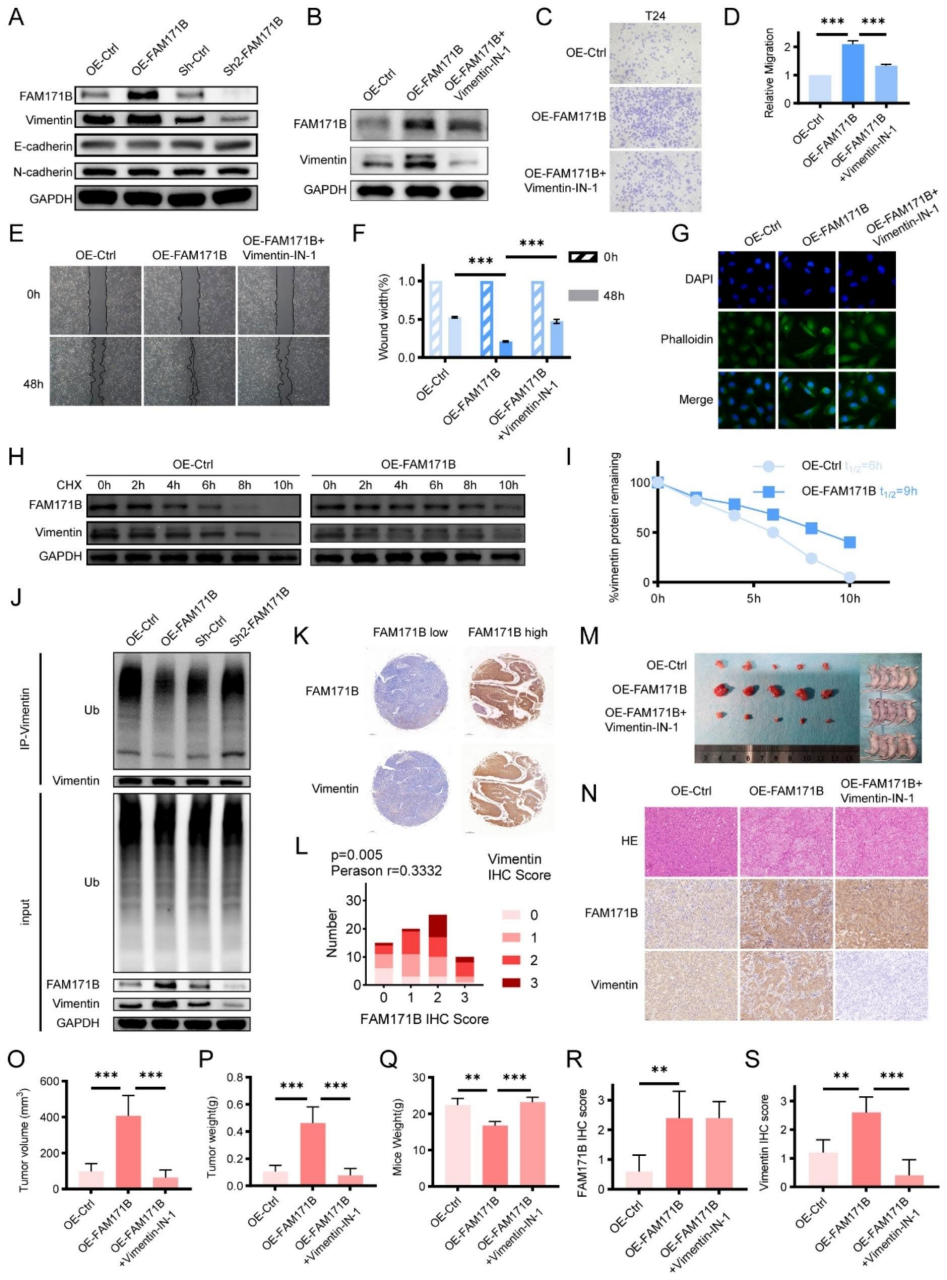
FAM171B stabilizes vimentin to promote the progression of bladder cancer. **A** Protein levels of FAM171B, vimentin, E-cadherin and N-cadherin in the FAM171B knockdown and overexpression T24 cell lines. **B** Protein levels of FAM171B and vimentin in the T24 cell lines in different groups. **C** Representative transwell images of T24 cells in different groups. **D** Statistical results of the number of invasive cells in each group of T24 cells. **E** Representative images of migration in T24 cells in different groups. **F** Statistical results of the number of migrated cells in each group of T24 cells. **G** Pictures of T24 cells in different groups. **H** Protein levels of FAM171B and vimentin in the T24 cells with or without FAM171B overexpression after treatmented with CHX (20 μg/ml) for various times. **I** Remaining vimentin protein level percentages in the T24 cells with or without FAM171B overexpression after treated with CHX for various times. **J** The western blot images showed the FAM171B regulated vimentin ubiquitination in T24 cells. Vimentin proteins were isolated from the FAM171B knockdown or overexpression T24 cells by Co-IP, and followed detected the ubiquitination of vimentin by western blot. input, whole cell lysate. **K** Representative IHC staining images of FAM171B and vimentin in FAM171B high or low expression group from tissue array. **L** Correlation analysis of IHC scores for FAM171B and vimentin from tissue array. **M** The pictures of tumors derived from human bladder cancer T24 cell line. **N** IHC staining pictures of FAM171B and vimentin expression and HE staining pictures in different groups. **O** The tumor volume in different groups. **P** The tumor weight in different groups. **Q** The average body weight of mice in different groups. **R** Analysis of FAM171B IHC score in tumors. **S** Analysis of vimentin IHC score in tumors

Vimentin is a protein marker for EMT, a process in which cancer cells acquire a migratory and invasive phenotype [22]. Therefore, we examined the influence of FAM171B on the EMT process in T24 cells. We observed a clear increase in vimentin protein levels upon FAM171B overexpression, while vimentin levels decreased in FAM171B knockdown cells compared to control cells. However, no significant changes in the expression of E-cadherin and N-cadherin were detected in either FAM171B overexpression or knockdown T24 cells (Fig. 5A). Additionally, through immunohistochemical (IHC) staining analysis of tissue microarray samples, we found a positive correlation between the protein levels of FAM171B and vimentin (Fig. 5K and L). Previous results demonstrated the interaction between FAM171B and vimentin in bladder cancer cells. Western blot analysis revealed the increase in the vimentin protein level upon overexpression of FAM171B that could be reversed by treatment with Vimentin-IN-1, a vimentin inhibitor that induces degradation of vimentin (Fig. 5B). To investigate whether FAM171B enhances the invasion and migration of bladder cancer cells through vimentin, we conducted wound healing and transwell invasion assays in cells treated with Vimentin-IN-1. The results of the Transwell invasion assay demonstrated that Vimentin-IN-1 partially inhibited the increase in the invasiveness of T24 cells caused by FAM171B overexpression (Fig. 5C and D). Similarly, the results of the wound healing assay showed that Vimentin-IN-1 treatment reduced the increase in the migration rate of T24 cells induced by FAM171B overexpression (Fig. 5E and F). Stretched F-actin fibers and a spindle-shaped morphology are characteristic features of cells undergoing EMT [23]. The phalloidin staining results demonstrated that efficient overexpression of FAM171B led to morphological changes in bladder cancer cells, which transitioned from an epithelial morphology to a mesenchymal morphology (Fig. 5G). We observed no significant change in vimentin mRNA levels in response to FAM171B overexpression or knockdown (Fig. S5A). Moreover, there was no correlation between the mRNA expression levels of FAM171B and vimentin in the TCGA-BLCA cohort (Fig. S5B). These results excluded the possibility of the transcriptional regulation of vimentin by FAM171B. Additionally, among the FAM171B-interacting proteins identified through IP-MS, we did not detect any known proteins associated with vimentin. Consequently, we hypothesized that FAM171B may enhance the stability of vimentin protein through post-translational mechanisms. Next, we inhibited protein synthesis in bladder cancer cells using cycloheximide (CHX), and subsequently measured the level of remaining vimentin protein by western blotting. After 10 h of CHX treatment, the level of vimentin in FAM171B-overexpressing cells was higher than that in control cells (Fig. 5H). Upon analysis, it was observed that overexpression of FAM171B extended the half-life of the endogenous vimentin protein from 6 to 9 h, suggesting that FAM171B can stabilize the vimentin protein (Fig. 5I). Subsequently, we investigated whether FAM171B affected the ubiquitination of vimentin in T24 cells. The results demonstrated that FAM171B overexpression led to a reduction in the level of vimentin ubiquitination, while FAM171B knockdown resulted in an increase in the level of ubiquitination (Fig. 5J). Similar results were obtained with MB49 mouse bladder cancer cells (Fig. S6). To further validate the role of FAM171B/vimentin in vivo, we established a mouse subcutaneous xenograft model using T24 cells (Fig. 5M). IHC staining analysis of the xenograft tumors in mice demonstrated a significant increase in FAM171B expression in the OE-FAM171B group, confirming the successful establishment of the model (Fig. 5N and R). Furthermore, FAM171B upregulated vimentin expression in vivo, and this effect was reversed by Vimentin-IN-1 treatment, similar to the in vitro findings (Fig. 5N and S). In mouse tumors, overexpression of FAM171B resulted in increases in the tumor volume and mass, while treatment with Vimentin-IN-1 effectively mitigated this effect (Fig. 5O and P). Additionally, in terms of mouse weight, treatment with Vimentin-IN-1 partially alleviated the weight loss caused by FAM171B overexpression (Fig. 5Q). In summary, these results confirm that FAM171B contributes to bladder cancer progression by stabilizing vimentin both in vivo and in vitro.

### FAM171B regulates CCL2 via HNRNPU and promotes TAM migration and infiltration in bladder cancer

**Fig. 6:**
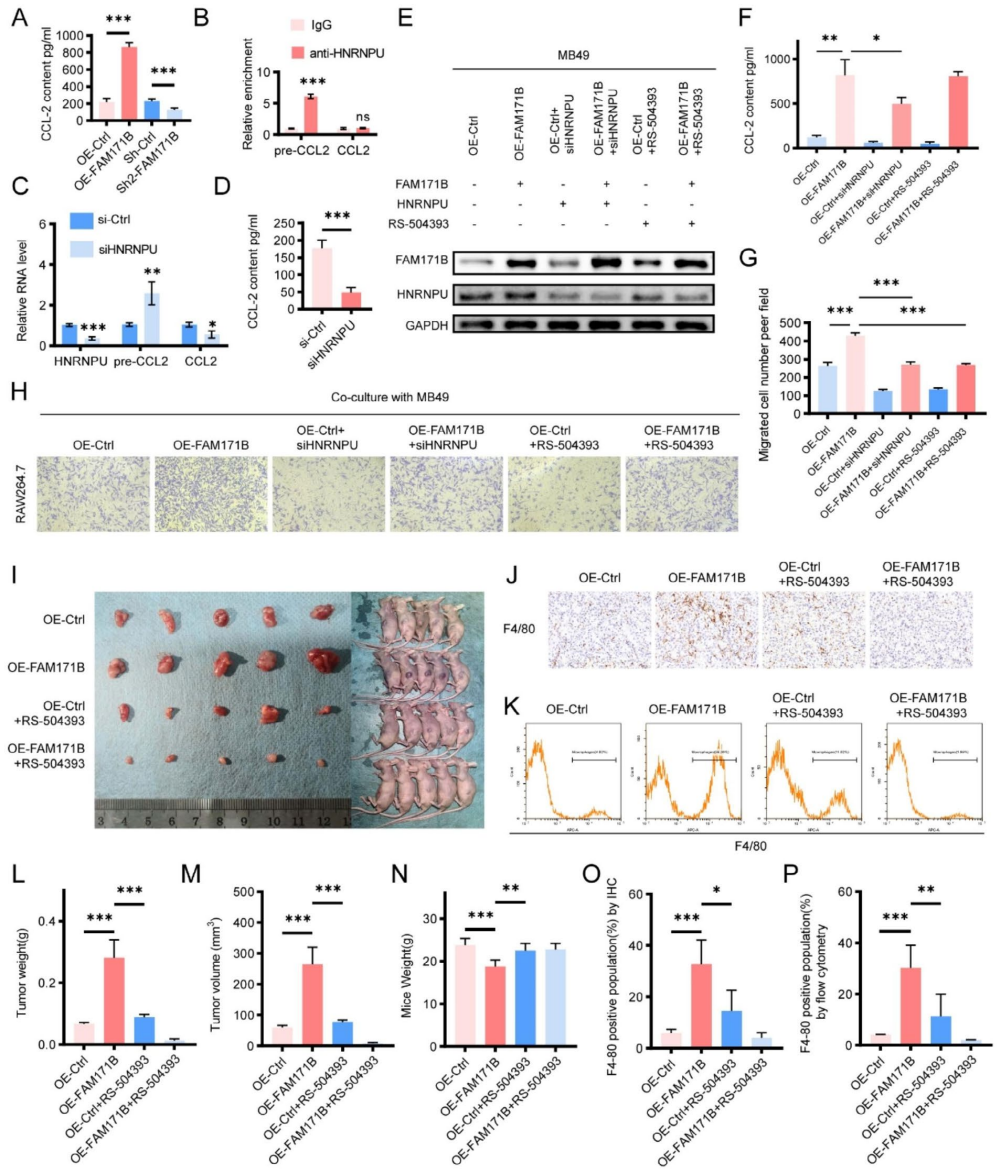
FAM171B regulates CCL2 via HNRNPU and promotes TAM migration and infiltration in bladder cancer. **A** ELISA analysis of CCL2 secretion levels in MB49 cells with FAM171B overexpression or knockdown. **B** RIP results show that HNRNPU can immunoprecipitate with CCL2 precursor RNA. **C** qRT-PCR analysis of the relative RNA levels of precursor CCL2 and CCL2 in MB49 cells with HNRNPU knockdown or control. **D** ELISA analysis of CCL2 secretion levels in MB49 cells with HNRNPU knockdown or control. **E** Protein levels of FAM171B and HNRNPU in the MB49 cell lines in different groups. **F** ELISA analysis of CCL2 secretion levels in MB49 cells in different groups. **G** Analysis of migration of RAW264.7(mouse macrophages) toward MB49 cells in different groups. **H** Representative images of migration of RAW264.7(mouse macrophages) toward MB49 cells in co-culture chambers. **I** The pictures of tumors derived from mouse bladder cancer MB49 cell line. **J** IHC staining pictures of F4/80(macrophage marker) expression in different groups. **K** Representative images of flow cytometry used to evaluate F4/80 positive subpopulations. **L** The tumor weight in different groups. **M** The tumor volume in different groups. **N** The average body weight of mice in different groups. **O** Analysis of F4/80 positive population by IHC. **P** Analysis of F4/80 positive population by flow cytometry

Our previous results demonstrated that HNRNPU is an important interacting protein of FAM171B in bladder cancer cells. Another study indicated the RNA-binding protein HNRNPU can enhance CCL2 expression [24]. Therefore, we conducted in vivo and in vitro experiments to investigate the effects of FAM171B and HNRNPU on CCL2 expression in bladder cancer cells. The ELISA results revealed that overexpression of FAM171B increased CCL2 secretion and knockdown of FAM171B decreased CCL2 secretion in MB49 mouse bladder cancer cells (Fig. 6A). The results of RIP experiments confirmed the binding of HNRNPU to CCL2 precursor RNA in MB49 cells (Fig. 6B). Consistent with previous findings, knockdown of HNRNPU in MB49 cells increased the level of CCL2 precursor RNA and reduced the level of CCL2 RNA (Fig. 6C). The ELISA results also confirmed that knockdown of HNRNPU decreased CCL2 secretion in MB49 cells (Fig. 6D). To investigate whether FAM171B and HNRNPU affect CCL2 secretion through a common pathway or distinct pathways, we performed separate interventions of HNRNPU knockdown or RS 504,393 (a CCR2 inhibitor) treatment in FAM171B-overexpressing MB49 cells (Fig. 6E). The ELISA results showed that knockdown of HNRNPU attenuated the increase in CCL2 secretion caused by FAM171B overexpression (Fig. 6F). CCL2 is a well-known chemotactic factor that induces specific chemotaxis of monocytes and macrophages [25]. To explore the effect of FAM171B and HNRNPU on macrophage chemotaxis, we cocultured RAW264.7 mouse macrophages with different groups of MB49 mouse bladder cancer cells. The results of the chemotaxis assays demonstrated that FAM171B overexpression enhanced the chemotaxis of macrophages towards tumor cells in the coculture system, while HNRNPU knockdown and CCR2 inhibition partially attenuated this effect (Fig. 6G and H). Similar results were obtained with T24 human bladder cancer cells cocultured with THP-1-derived human macrophages (Fig. S7). To further validate the effect of FAM171B/CCL2 modulation in vivo, we established a mouse subcutaneous transplantation tumor model using MB49 bladder cancer cells (Fig. 6I). The immunohistochemical analysis (Fig. 6J and O) and flow cytometry (Fig. 6K and P) results confirmed that FAM171B overexpression significantly increased macrophage infiltration in tumor tissues and that CCR2 inhibitor treatment effectively suppressed this effect. Importantly, CCR2 inhibitors also partially reversed the increase in tumor growth (Fig. 6L and M) and alleviated the weight loss (Fig. 6N) caused by FAM171B overexpression. In conclusion, these experiments demonstrate that FAM171B promotes CCL2 secretion via HNRNPU, resulting in macrophage infiltration and tumor progression.

### FAM171B regulates CCL2 expression to enhance TAM polarization towards the M2 phenotype

**Fig. 7:**
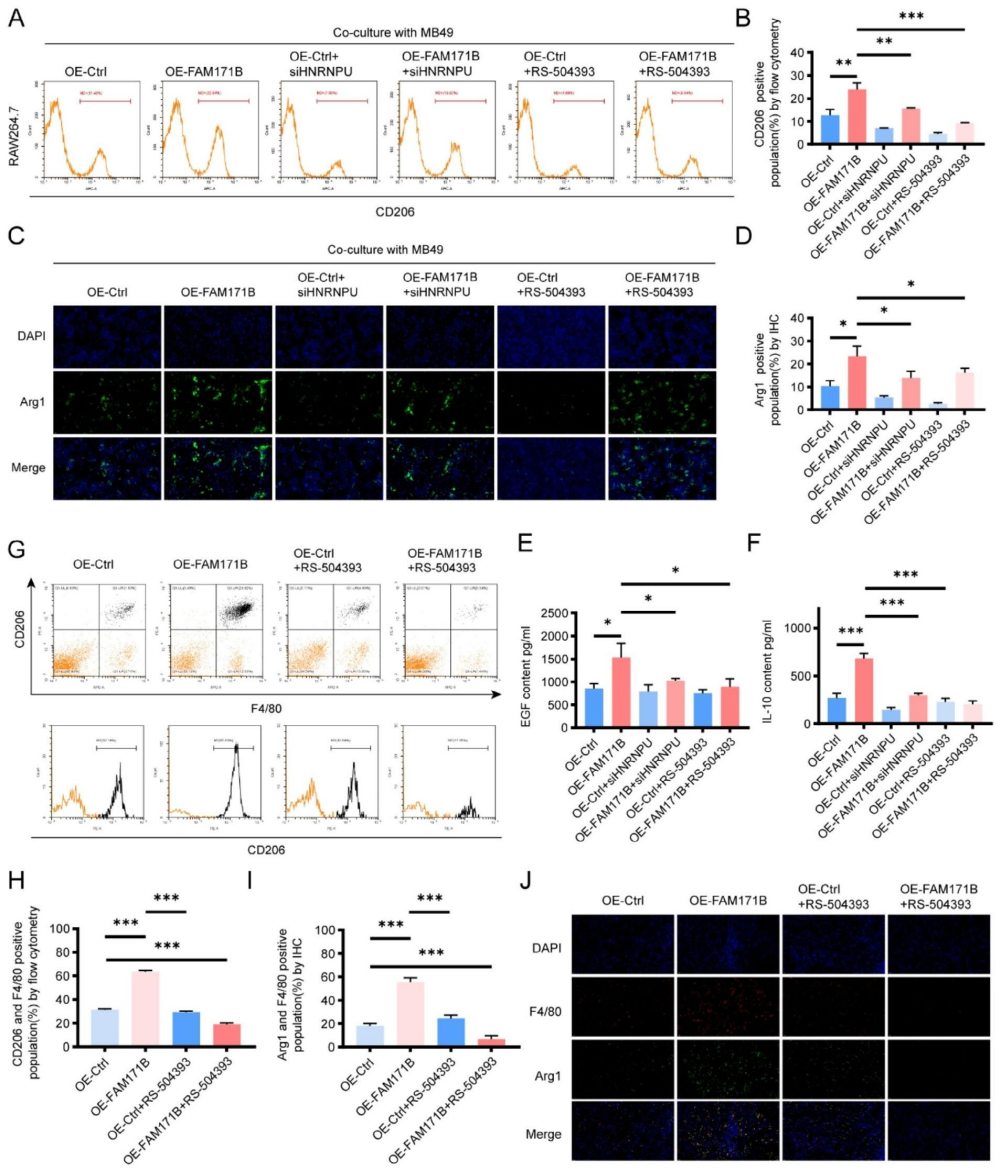
FAM171B regulates CCL2 expression to enhance TAM polarization towards the M2 phenotype. **A** Representative images of flow cytometry used to evaluate CD206 positive subpopulations in RAW264.7 co-cultured with MB49 cells in different groups. **B** Analysis of CD206 positive population by flow cytometry. **C** Representative images of immunostaining of Arg1 (M2 marker) in RAW264.7 co-cultured with MB49 cells. **D** Analysis of Arg1 positive population by immunostaining. **E** Analysis of EGF (M2 marker) secretion levels in RAW264.7 co-cultured with MB49 cells in different groups. **F** Analysis of IL-10 (M2 marker) secretion levels in RAW264.7 co-cultured with MB49 cells in different groups. **G** Representative images of flow cytometry used to evaluate CD206 and F4/80 positive subpopulations in MB49 subcutaneous graft tumor in different groups. **H** Analysis of CD206 and F4/80 positive population in MB49 subcutaneous graft tumor by flow cytometry. **I** Analysis of Arg1 and F4/80 positive population in MB49 subcutaneous graft tumor by immunostaining. **J** Representative images of immunostaining of Arg1 (M2 marker) and F4/80 (macrophage marker) in MB49 subcutaneous graft tumor in different groups

The M2 phenotype is a dominant characteristic of tumor-associated macrophages in the TME and promotes tumor progression [26]. The CCL2-CCR2 pathway is one of the pathways that facilitates M2 polarization of macrophages [27, 28]. Therefore, we investigated the impact of FAM171B/CCL2 modulation on bladder cancer-associated macrophage polarization both in vivo and in vitro using double immunofluorescence staining and flow cytometry. Flow cytometry analysis revealed an increase in the proportion of CD206-positive RAW264.7 macrophages (M2 marker) when cocultured with FAM171B-overexpressing MB49 cells compared to control cells. Knockdown of HNRNPU and treatment with the CCR2 inhibitor partially reversed the increase in CD206 positivity induced by FAM171B overexpression (Fig. 7A and B). In the IHC, we utilized another M2 macrophage marker, Arg1, and obtained similar results (Fig. 7C and D). Furthermore, we observed significant increases in the secretion of EGF and IL-10, both of which are M2 macrophage markers, in the medium of the coculture system, as measured by ELISA (Fig. 7E and F). Consistent findings were observed when T24 human bladder cancer cells were cocultured with THP-1-derived human macrophages (Fig. S8). Interestingly, THP-1-derived macrophages exhibited enhanced aggregation after coculture with FAM171B-overexpressing T24 cells. To further validate the roles of FAM171B and CCL2 in vivo, we examined the ratio of M2 macrophages in mouse transplanted tumors. In line with the results of the in vitro experiments, the proportion of M2 macrophages in the subcutaneous tumors was increased in the FAM171B overexpression group of mice, and treatment with the CCR2 inhibitor significantly reversed this effect of FAM171B overexpression (Fig. 7G, H, I and J). Taken together, these experimental results confirm that FAM171B has the ability to promote M2 polarization of TAM in the TME through the CCL2 pathway.

## Discussion

Given the current clinical failures in the treatment of patients with advanced metastatic bladder cancer, it remains imperative to investigate the molecular mechanisms underlying the progression of this disease. Here, we identified FAM171B as a significant factor driving bladder cancer progression. We observed positive correlations between FAM171B expression and worse overall survival (OS), progression-free survival (PFS), and disease-specific survival (DSS) outcomes in bladder cancer patients. Additionally, FAM171B was associated with advanced clinicopathological stages in these patients, implying that FAM171B may promote the progression of local invasion and distant metastasis in bladder cancer. Notably, pancancer analysis revealed that FAM171B is also linked to poor prognosis in other malignancies, including kidney renal papillary cell carcinoma and stomach adenocarcinoma. These results demonstrate that FAM171B is an independent predictor of bladder cancer progression with an important cancer-promoting mechanism.

FAM171B is a recently discovered protein, and there is limited knowledge regarding its structure, cellular localization, and biological function. Bioinformatic analysis revealed that the FAM171B gene contains 8 exons, and undergo splicing to generate three distinct isoforms. Currently, there is only one validated anti-FAM171B antibody on the market that can be used for western blotting. In our western blot analysis using the mouse bladder cancer cell line MB49 and the human bladder cancer cell line T24, we detected two isoforms of FAM171B using this antibody. Regarding the subcellular localization of FAM171B, a study by Quan Tran suggested that although FAM171B is predicted to contain a putative bipartite nuclear localization signal, it is expressed predominantly in the cytoplasm of neuronal cells [16]. However, in our experiments, FAM171B was found to be expressed in both the cytoplasm and the nucleus of human and mouse bladder cancer cell lines. These results indicate that while FAM171B is expressed widely throughout the body, it exhibits cell context-specific isoform expression and localization, which may contribute to its diverse functions.

We identified vimentin as an important interacting protein of FAM171B in the cytoplasm of bladder cancer cells. Vimentin, an intermediate filament protein associated with EMT, has been reported to be linked with increased tumor growth, invasion, and poor prognosis in bladder cancer [7, 8]. Patients with NMIBC exhibiting higher vimentin expression had poorer DFS, with expression increasing with progression from NMIBC to MIBC and metastasis [8]. Studies by Baumgart et al. revealed that vimentin expression was predominantly detected in invasive bladder cancer (31% in MIBC vs. 7% in NMIBC) and positively correlated with tumor grade and stage [29]. Various factors have been reported to regulate vimentin levels [23, 30, 31]. Stabilization of vimentin proteins can play a crucial role in cancer cell adhesion, invasion, and survival, leading to local and distant metastasis [23, 32]. Notably, vimentin is not typically expressed in normal urothelial epithelium or epithelial tumors [7]. This supports the hypothesis that targeting vimentin to inhibit EMT would have limited adverse effects on nonmalignant cells while effectively impeding cancer progression to metastatic disease. Multiple lines of evidence have indicated that ubiquitination may represent a crucial post-translational modification involved in vimentin degradation [33, 34]. Our study demonstrated that FAM171B enhances the stabilization of vimentin by binding to vimentin and reducing its ubiquitination. We found that FAM171B-induced migration, invasion, and EMT could be blocked by treatment with a vimentin inhibitor, suggesting that FAM171B promotes these processes in a manner at least partially dependent on vimentin. Our study uncovered a novel mechanism for regulating vimentin at the protein level in bladder cancer and partially explains the relationship of FAM171B with poor prognosis in bladder cancer. Metastasis remains a major obstacle to effective bladder cancer therapy. In this study, we revealed that FAM171B promotes bladder cancer progression through its interaction with vimentin, highlighting FAM171B as a critical regulator of cancer metastasis. Targeting FAM171B to reduce vimentin levels could offer a promising strategy for preventing metastasis in patients with advanced bladder cancer. Cancer associated fibroblasts (CAFs) are the main stromal cell type and implicated in the progression and poor prognosis of bladder cancer [35]. Since vimentin is also considered a marker for fibroblasts, we investigated the connection between FAM171B and fibroblast infiltration. However, our experiments did not reveal an association between FAM171B and CAF infiltration in bladder cancer.

FAM171B plays a crucial role as an immunomodulatory factor in tumors. Regarding bladder cancer, RNA sequencing of T24 cells revealed that FAM171B is associated primarily with immune functions, including cytokine activity and the immune response. Moreover, the results of pancancer immune infiltration analysis across 33 tumor types demonstrated distinct immunomodulatory effects of FAM171B on different types of immune cells. Macrophages were found to be the most abundant immune cells in the MIBC microenvironment [36]. TAMs have been extensively studied for their interactions with tumor cells [15]. Accumulating evidence suggests that TAM accumulation influences angiogenesis and tumor cell proliferation, leading to a worse prognosis in bladder cancer patients [37]. CCL2, an important chemokine for macrophage, is highly expressed in and secreted by cancer cells. CCL2 regulates the TME by enhancing the infiltration of TAMs, which are derived from circulating monocytes in vessels [38]. In addition, several studies have demonstrated that CCL2 promotes M2 polarization of TAMs, thereby enhancing the immunosuppressive properties of the TME [27, 28, 39]. In this study, we found that FAM171B-induced CCL2 modulated the TME by promoting the infiltration and M2 polarization of TAMs, ultimately leading to tumor progression. Importantly, inhibition of CCR2 partially attenuated FAM171B-induced tumor progression. These results uncovered a new mechanism underlying the interaction between bladder cancer cells and TAMs and partially explained the association of FAM171B with poor prognosis in terms of the immune microenvironment. In the tumor immune microenvironment, multiple functionally distinct immune cells form a complex network. Several studies have previously reported that M2-TAMs contribute to immunosuppression, in part by inhibiting CD8 + T cell infiltration within the tumor microenvironment [27, 40]. CD8 + T cells are recognized as the primary cytotoxic immune cells responsible for tumor clearance. In our study, both bioinformatics and immunofluorescence analyses revealed a negative correlation between FAM171B and CD8 + T cell infiltration, suggesting that FAM171B may exacerbate immunosuppression and facilitate tumor progression. The immunomodulatory effects of FAM171B on other immune cells may also involve CCL2. While CCL2 primarily influences monocyte macrophages, it is also implicated in the recruitment of various other cell types, including natural killer cells, dendritic cells, and neutrophils. The direct and indirect effects of FAM171B on other immune cells in addition to macrophages still need to be explored using new experimental models in the future. Furthermore, the pancancer immune analysis has revealed that FAM171B plays extensive and important immunomodulatory roles across various tumor types. Beyond its implications in bladder cancer, FAM171B seems to exert substantial immunomodulatory effects in digestive tumors, including colon adenocarcinoma, liver hepatocellular carcinoma, and pancreatic adenocarcinoma. Consequently, the immunomodulatory functions of FAM171B in other malignancies warrant comprehensive investigations.

We found that FAM171B upregulates CCL2 expression and that these functions are mediated at least partially through its interaction with HNRNPU. We identified HNRNPU as another important interacting protein of FAM171B in the nucleus of bladder cancer cells. HNRNPU belongs to the heterogeneous nuclear ribonucleoprotein (hnRNP) family, which are RNA-binding proteins involved in the regulation of alternative splicing [41]. HNRNPU is highly expressed in most tumors, and several studies have indicated that HNRNPU promotes tumor progression through alternative splicing [42–44]. Notably, a previous study showed that HNRNPU acts as an RNA-binding protein that positively regulates CCL2 mRNA levels [24]. The discovery of FAM171B’s interaction with HNRNPU led us to hypothesize that FAM171B might be involved in the regulation of CCL2 pre-mRNA alternative splicing through its interaction with HNRNPU. We observed that FAM171B and HNRNPU synergistically regulated the production of CCL2 mRNA through alternative splicing of CCL2 pre-mRNA. Knockdown of HNRNPU blocked the effects of FAM171B on the upregulation of CCL2 expression and on the chemotaxis and M2 polarization of macrophages. Overall, we found a molecular mechanism of CCL2 overexpression in the bladder cancer and showed that treatment targeting FAM171B would be an effective approach for bladder cancer patients with high TAM infiltration. Considering the broad functions of HNRNPU, it is plausible that FAM171B may perform other important functions in tumors by synergizing with HNRNPU. Further research is needed to explore the comprehensive role of FAM171B and its interaction with HNRNPU in cancer.

Chemotherapy often results in the development of drug resistance, ultimately leading to treatment failure. Previous studies have suggested that FAM171B might promote oxaliplatin resistance in colon cancer [18]. Our present study revealed that high expression of FAM171B is associated with reduced sensitivity to several commonly used chemotherapeutic agents in bladder cancer. The underlying mechanisms warrant further exploration in future. Immunotherapy has emerged as a crucial treatment modality for bladder cancer. However, similar to conventional chemotherapy, the development of resistance to immunotherapies remains a significant challenge. The infiltration of immunosuppressive cells, such as M2 tumor-associated macrophages, is a key contributor to the failure of immunotherapy. Modulating the tumor immune microenvironment by targeting FAM171B may represent a strategy to enhance the effectiveness of immunotherapy in bladder cancer. On the other hand, both targeting vimentin and the CCL2-CCR2 axis show promise as therapeutic strategies for bladder cancer. It is worth noting that discontinuing CCL2 inhibition can worsen tumor metastasis, although the mechanism remains unclear [45]. Our experimental findings demonstrated the impact of FAM171B on both vimentin and the CCL2-CCR2 axis, which significantly influences the progression of bladder cancer. Therefore, targeting FAM171B constitutes a potentially effective approach for bladder cancer treatment.

## Conclusion

**Fig. 8:**
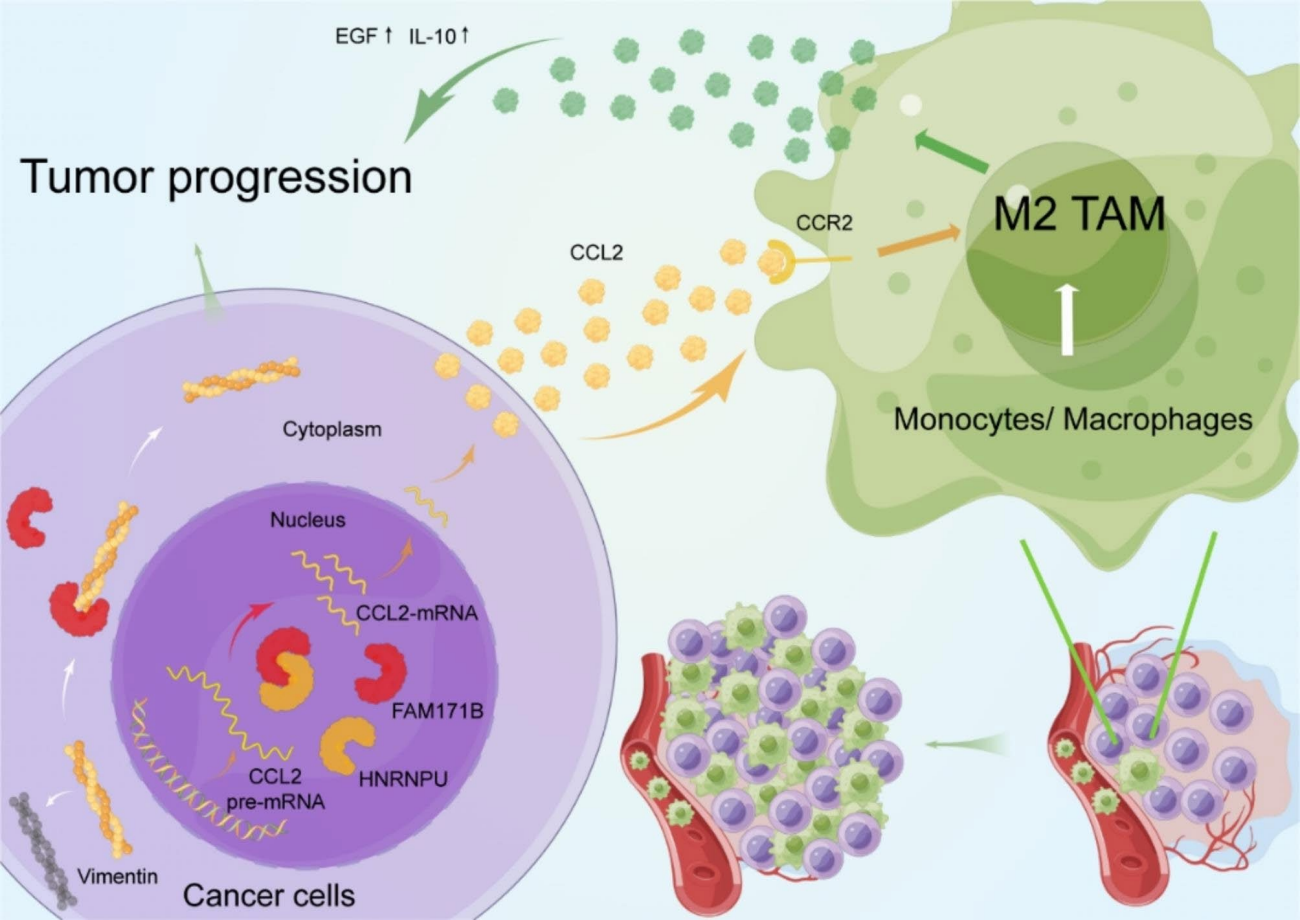
Schematic illustration. Schematic illustration of the functional roles of FAM171B in stabilizing vimentin and enhancing M2-TAM infiltration by HNRNPU/CCL2 and leading the malignant progression of bladder cancer

In this study, we identified FAM171B as a potent factor promoting bladder cancer progression that is positively correlated with advanced cancer stages and poor prognosis. Furthermore, FAM171B exerts a stabilizing effect by interacting with vimentin and enhancing its stability, leading to the tumor progression. Moreover, FAM171B enhances CCL2 mRNA splicing by interacting with HNRNPU, ultimately resulted in the recruitment of tumor-associated macrophages (TAMs) and their polarization towards the M2 phenotype. Based on our comprehensive findings, FAM171B has great potential as a biomarker for bladder cancer diagnosis and therapeutic interventions (Fig. 8).

### Electronic Supplementary Material

Below is the link to the electronic supplementary material.

Supplementary Material 1

## Data Availability

The authors declare that all the data supporting the findings in this study are available in this study and are available from the corresponding author through reasonable request.

## Abbreviations

BLCA: Bladder Urothelial Carcinoma
CCL2: Chemokine (C-C motif) ligand 2
CCR2: Chemokine C-C-Motif Receptor 2
CHX: Cycloheximide
DFS: Disease-free survival
DSS: Disease-specific survival
eggNOG: Evolutionary gene genealogy Non-supervised Orthologous Groups
EMT: Epithelial-to-mesenchymal transition
FAM171B: Family with sequence similarity 171B
FBS: Fetal Bovine Serum
GEO: Gene Expression Omnibus
GO: Gene Ontology
HE: Hematoxylin-eosin
HNRNPU: Heterogeneous nuclear ribonucleoprotein U
IF: Immunofluorescent
IHC: Immunohistochemistry
KEGG: Kyoto Encyclopedia of Genes and Genomes
MIBC: Muscle invasive bladder cancer
NMIBC: Non-muscle invasive bladder cancer
OS: Overall survival
PFS: Progression-free survival
PMA: Phorbol 12-myristate 13-acetate
qRT-PCR: Quantitative real-time polymerase chain reaction
RIP: RNA immunoprecipitation
shRNA: Short hairpin RNA
siRNA: Small interfering RNA
TAM: Tumor-associated macrophage
TCGA: The Cancer Genome Atlas
TME: Tumor microenvironment
VIM: vimentin
