# Supplementary material for: FAM171B stabilizes vimentin and enhances CCL2-mediated TAM infiltration to promote bladder cancer progression
Source: J Exp Clin Cancer Res. 2023 Nov 2;42:290. doi: 10.1186/s13046-023-02860-5 (PMC10621219; doi:10.1186/s13046-023-02860-5)
Supplement: Supplementary file 1 — Supplementary Material 1 [file 13046_2023_2860_MOESM1_ESM.docx]

**Figure S1**

**
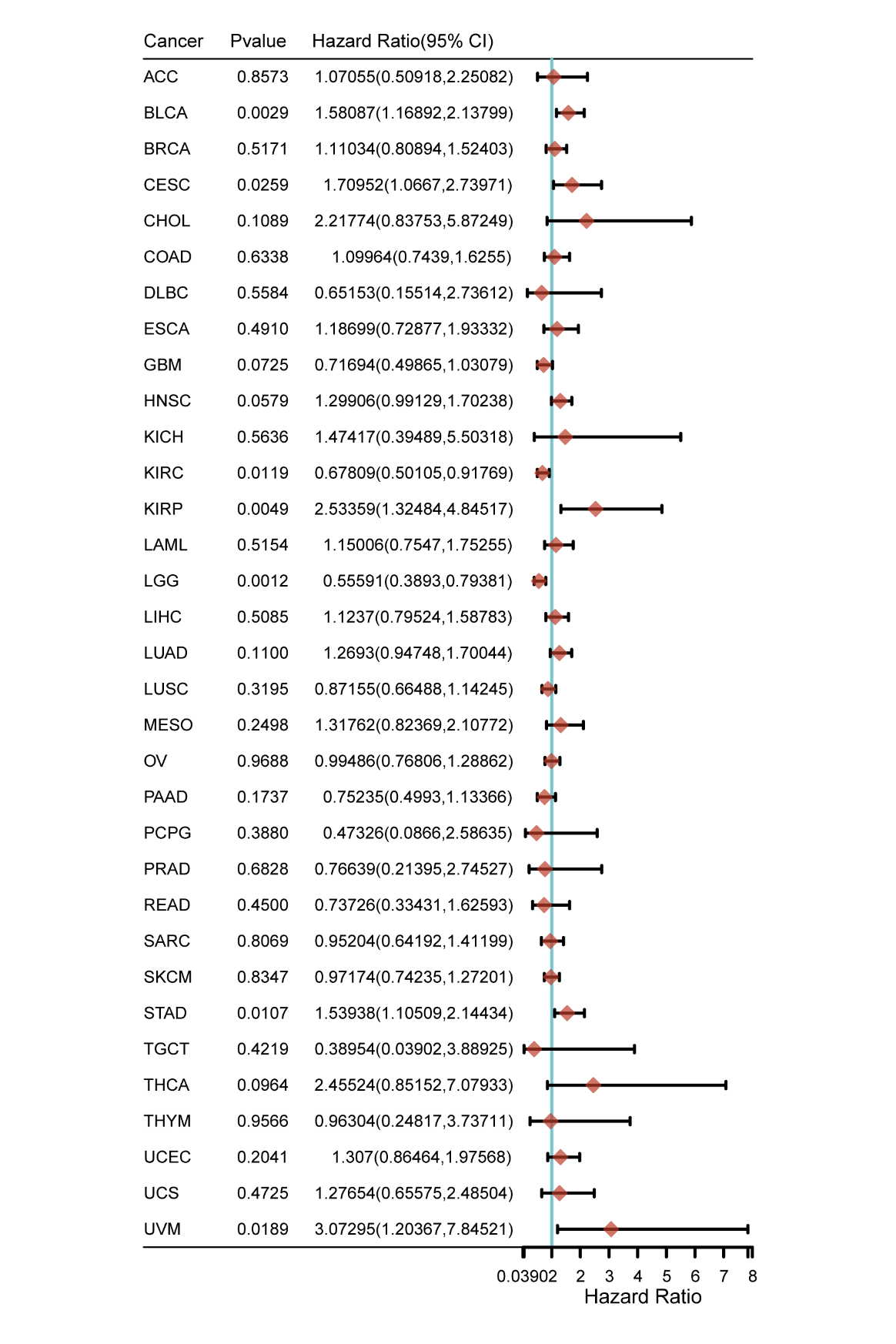
**

Prognostic analysis of FAM171B in pan-cancer. ACC, Adrenocortical carcinoma; BLCA, Bladder Urothelial Carcinoma; BRCA, Breast invasive carcinoma; CESC, Cervical squamous cell carcinoma and endocervical adenocarcinoma; CHOL, Cholangio carcinoma; COAD, Colon adenocarcinoma; DLBC, Lymphoid Neoplasm Diffuse Large B-cell Lymphoma; ESCA, Esophageal carcinoma; GBM, Glioblastoma multiforme; HNSC, Head and Neck squamous cell carcinoma; KICH, Kidney Chromophobe; KIRC, Kidney renal clear cell carcinoma; KIRP, Kidney renal papillary cell carcinoma; LAML, Acute Myeloid Leukemia; LGG, Brain Lower Grade Glioma; LIHC, Liver hepatocellular carcinoma; LUAD, Lung adenocarcinoma; LUSC, Lung squamous cell carcinoma; MESO, Mesothelioma; OV, Ovarian serous cystadenocarcinoma; PAAD, Pancreatic adenocarcinoma; PCPG, Pheochromocytoma and Paraganglioma; PRAD, Prostate adenocarcinoma; READ, Rectum adenocarcinoma; SARC, Sarcoma; SKCM, Skin Cutaneous Melanoma; STAD, Stomach adenocarcinoma; TGCT, Testicular Germ Cell Tumors; THCA, Thyroid carcinoma; THYM, Thymoma; UCEC, Uterine Corpus Endometrial Carcinoma; UCS, Uterine Carcinosarcoma; UVM, Uveal Melanoma;

**Figure S2**

**
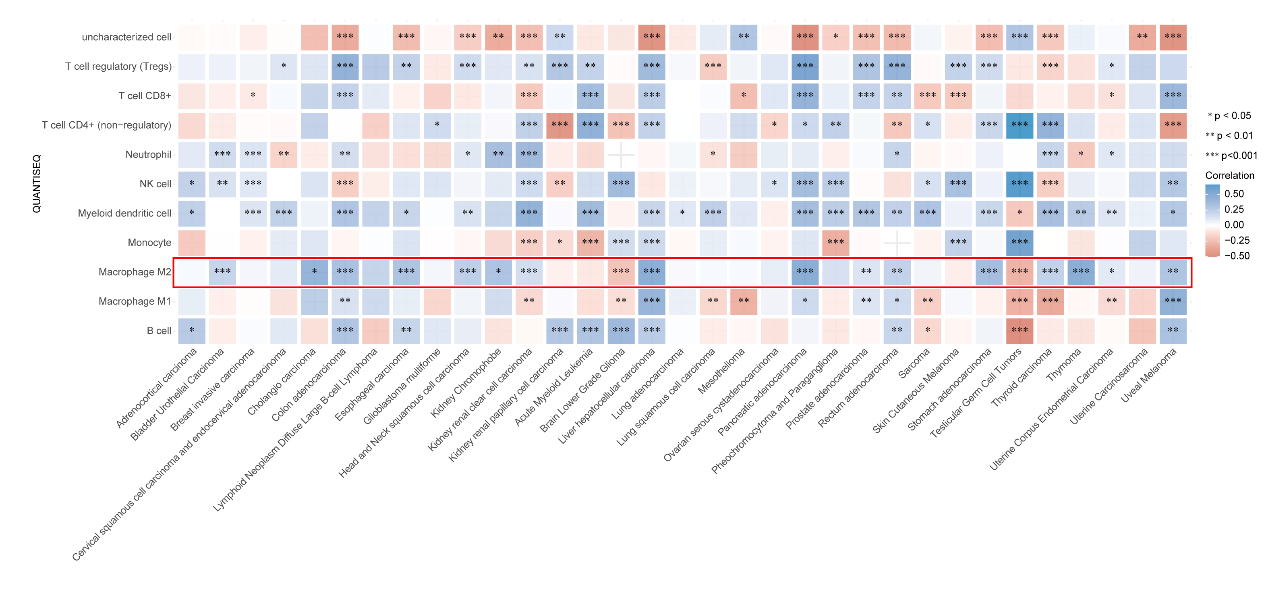
**

Immune analysis of FAM171B in pan-cancer using the QUANTISEQ algorithm. The abscissa represents different tumor tissues, and the ordinate represents different immune score of different immune cells. Different colors represent the correlation coefficients.

**Figure S3**

**
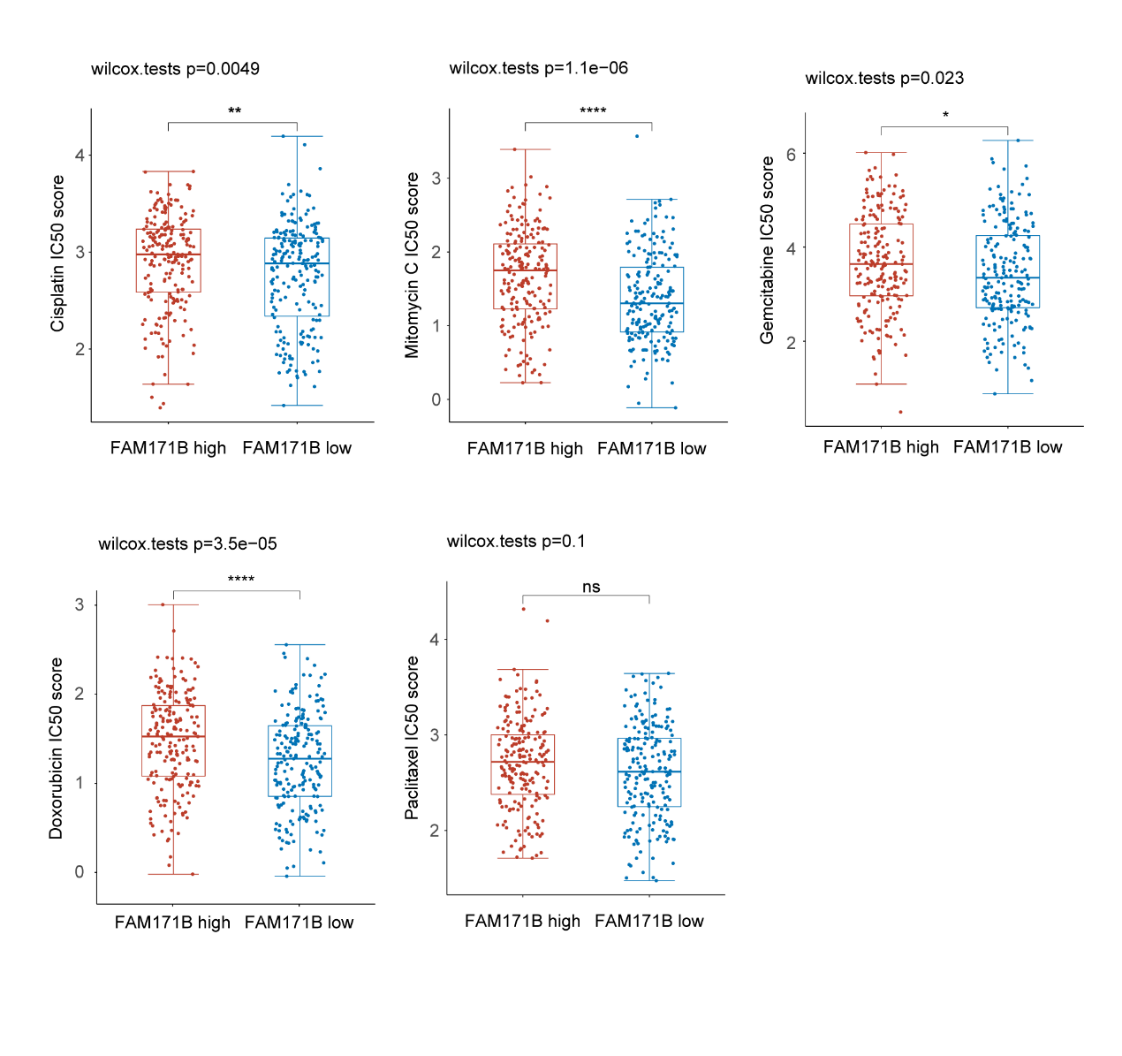
**

Drug sensitivity prediction of Cisplatin, Mitomycin, Gemcitabine, Doxorubicin and Paclitaxel in bladder cancer based on the Genomics of Drug Sensitivity in Cancer (GDSC). The abscissa represents FAM171B different expression groups, and the ordinate represents the distribution of the IC50 score. Higher IC50 scores represent poorer drug sensitivity.

**Figure S4**

**
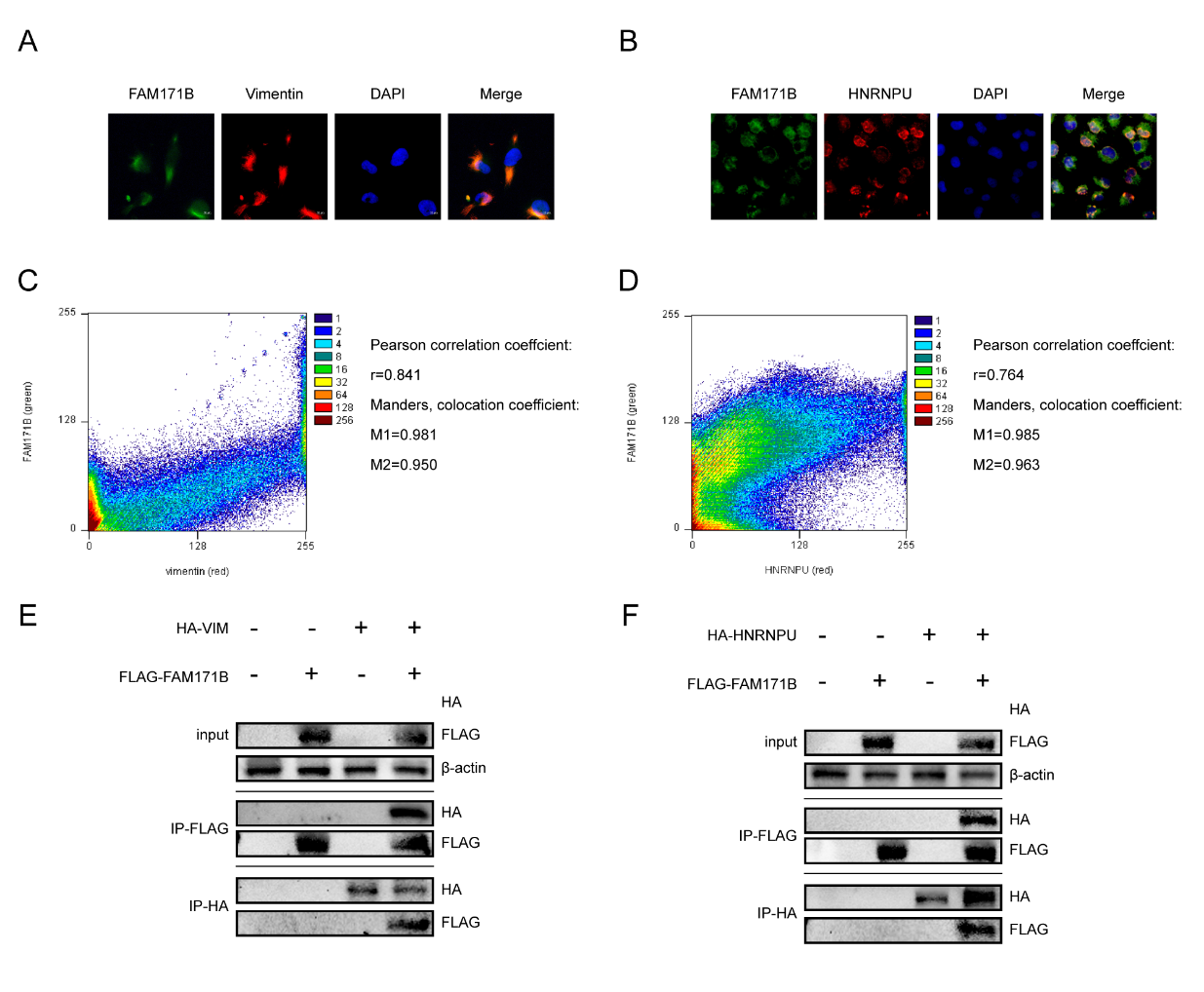
**

**A** Colocalization of FAM171B and vimentin was visualized by confocal microscope in MB49 cells. Cytoplasmic staining of FAM171B and vimentin was mostly merged together. **B** Colocalization of FAM171B and HNRNPU was visualized by confocal microscope in MB49 cells. Nuclear staining of FAM171B and HNRNPU was mostly merged together. **C** ImageJ was used for colocation analysis of FAM171B and vimentin. Pearson correlation analysis showed that r > 0.5. Manders’ colocation coefficient showed that M1 > 0.5 and M2 > 0.5. **D** ImageJ was used for colocation analysis of FAM171B and HNRNPU. Pearson correlation analysis showed that r > 0.5. Manders’ colocation coefficient showed that M1 > 0.5 and M2 > 0.5. **E, F** Co-IP assays were performed with anti-HA antibody beads or anti-FLAG antibody beads on cell lysates from MB49 cells transfected with Flag-FAM171B alone or together with the indicated HA-VIM(E) or HA-HNRNPU (F) constructs.

**Figure S5**


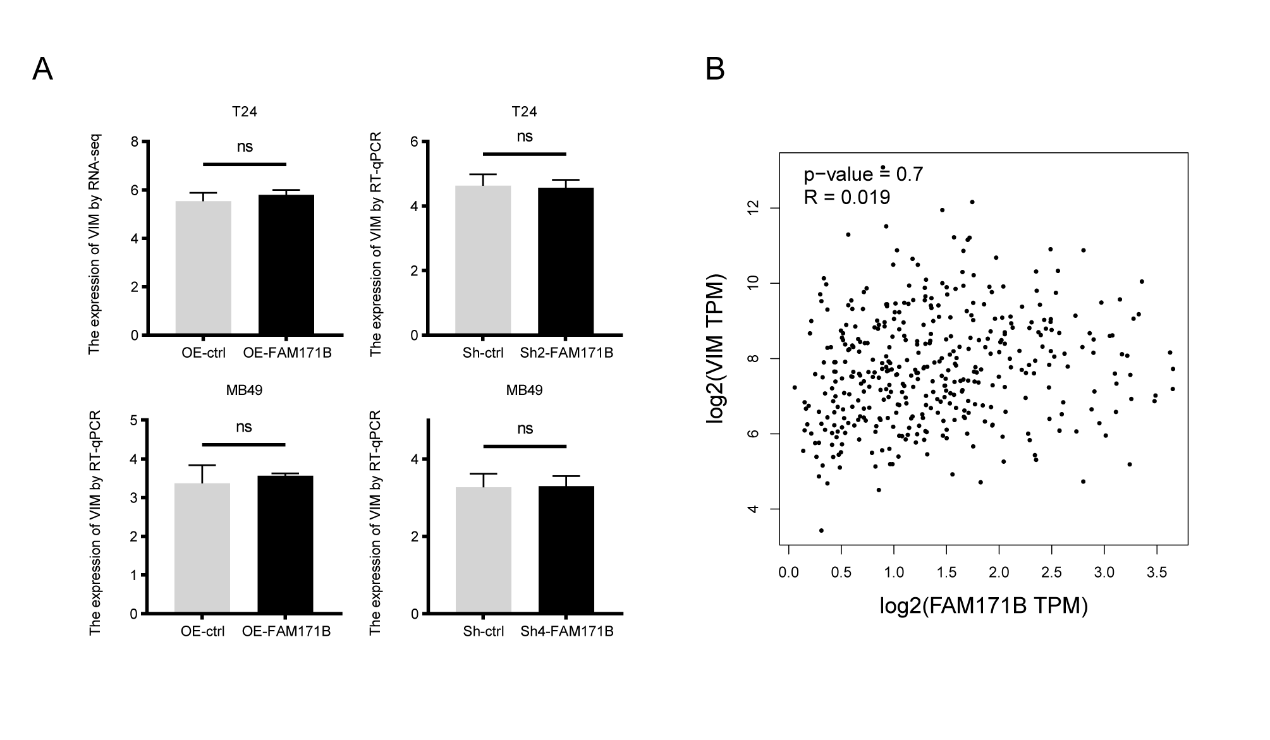


**A** The mRNA expression levels of VIM in the FAM171B knockdown and overexpression T24 and MB49 cell lines. **B** Correlation of mRNA expression levels of FAM171B and VIM in the TCGA-BLCA cohort.

**Figure S6**

**
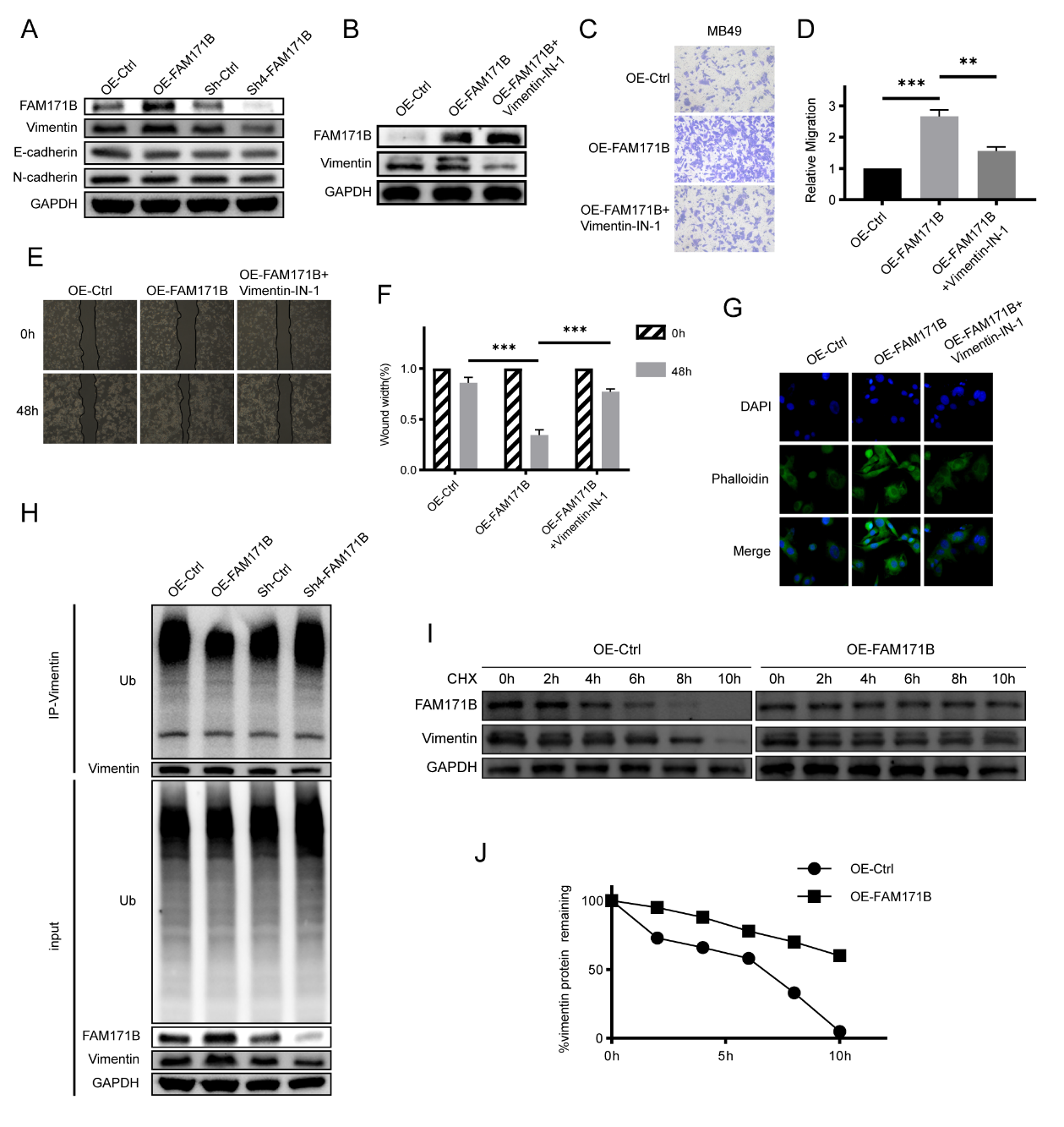
**

**A** Protein levels of FAM171B, Vimentin, E-cadherin and N-cadherin in the knockdown and overexpression MB49 cell lines. **B** Protein levels of FAM171B and vimentin in the MB49 cell lines in different groups. **C** Representative transwell images of MB49 cells in different groups. **D** Statistical results of the number of invasive cells in each group of MB49 cells. **E** Representative images of migration in MB49 cells in different groups. **F** Statistical results of the number of migrated cells in each group of MB49 cells. **G** Phalloidin staining pictures of MB49 cells in different groups. **H** The Western blot images showed the FAM171B regulated vimentin ubiquitination in MB49 cells. Vimentin proteins were isolated from the FAM171B knockdown or overexpression MB49 cells by Co-IP, and followed detected the ubiquitination of vimentin by Western blot. **I** Protein levels of FAM171B and vimentin in the MB49 cells with or without FAM171B overexpression after treatmented with CHX (20 μg/ml) for various times. **J** Remaining vimentin protein level percentages in the MB49 cells with or without FAM171B overexpression after treated with CHX (20 μg/ml) for various times.

**Figure S7**

**
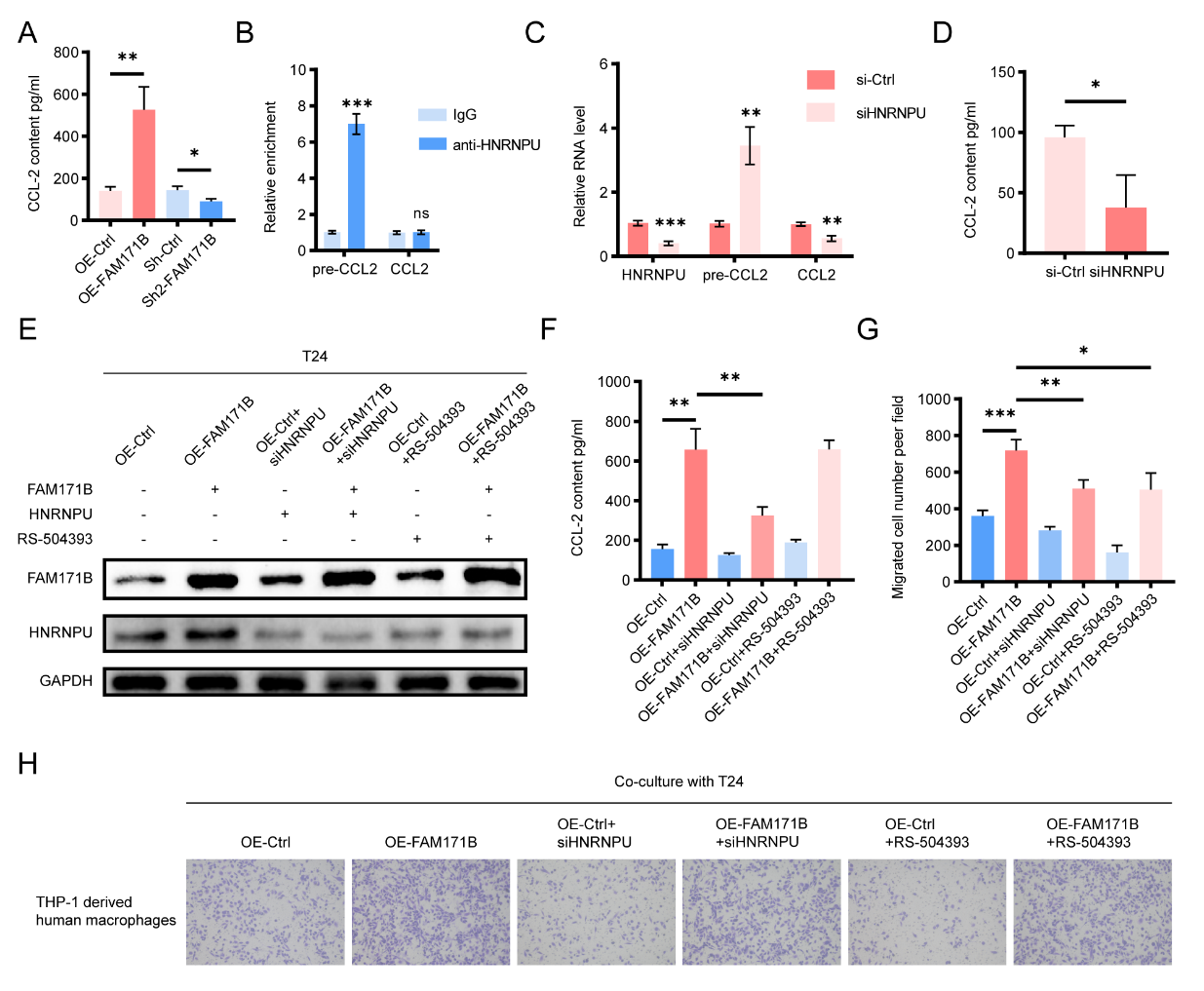
**

**A** ELISA analysis of CCL2 secretion levels in T24 cells with FAM171B overexpression or knockdown. **B** RIP results show that HNRNPU can immunoprecipitate with CCL2 precursor RNA in T24 cells. **C** qRT-PCR analysis of the relative RNA levels of precursor CCL2 and CCL2 in T24 cells with HNRNPU knockdown or control. **D** ELISA analysis of CCL2 secretion levels in T24 cells with HNRNPU knockdown or control. **E** Protein levels of FAM171B and HNRNPU in the T24 cell lines in different groups. **F** ELISA analysis of CCL2 secretion levels in T24 cells in different groups. **G** Analysis of migration of THP-1 derived human macrophages toward T24 cells in different groups. **H** Representative images of migration of THP-1 derived human macrophages toward T24 cells in co-culture chambers.

**Figure S8**

**
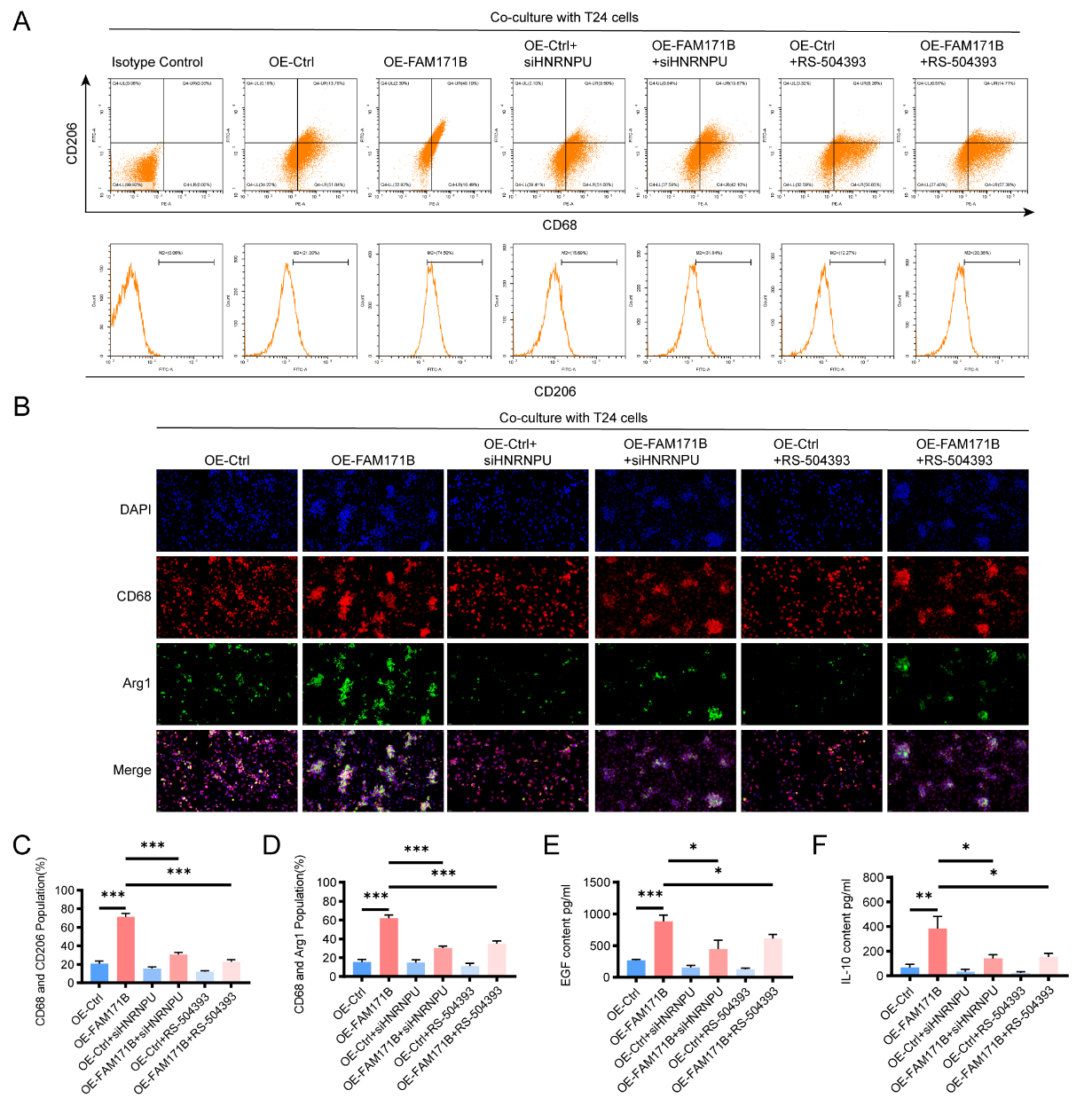
**

**A** Representative images of flow cytometry used to evaluate CD68(macrophage marker) and CD206(M2 marker) positive subpopulations in THP-1 derived human macrophages co-cultured with T24 cells in different groups. **B** Representative images of immunostaining of CD68 and Arg1 (M2 marker) in THP-1 derived human macrophages co-cultured with T24 cells in different groups. **C** Analysis of CD68 and CD206 positive population by flow cytometry. **D** Analysis of CD68 and Arg1 positive population by immunostaining. **E** Analysis of EGF secretion levels in THP-1 derived human macrophages co-cultured with T24 cells in different groups. **F** Analysis of IL-10 secretion levels in THP-1 derived human macrophages co-cultured with T24 cells in different groups.
